# Bio-Electrocatalytic Conversion of Food Waste to Ethylene via Succinic Acid as the Central Intermediate

**DOI:** 10.1021/acscatal.2c02689

**Published:** 2022-10-18

**Authors:** Christian M. Pichler, Subhajit Bhattacharjee, Erwin Lam, Lin Su, Alberto Collauto, Maxie M. Roessler, Samuel J. Cobb, Vivek M. Badiani, Motiar Rahaman, Erwin Reisner

**Affiliations:** † Yusuf Hamied Department of Chemistry, University of Cambridge, Lensfield Road, CB2 1EWCambridge, U.K.; ‡ Department of Chemistry and Centre for Pulse EPR Spectroscopy (PEPR), Imperial College, London Molecular Sciences Research Hub, White City Campus, Wood Lane, LondonW12 0BZ, U.K.; § Institute for Applied Physics, Vienna University of Technology, Wiedner Hauptstraße 8-10, A-1040Vienna, Austria; ∥ Centre of Electrochemistry and Surface Technology, Viktor Kaplan Straße 2, A-2700Wiener Neustadt, Austria

**Keywords:** decarboxylation reaction, bio-electrochemistry, waste conversion, ethylene production, circular economy, sustainable resources

## Abstract

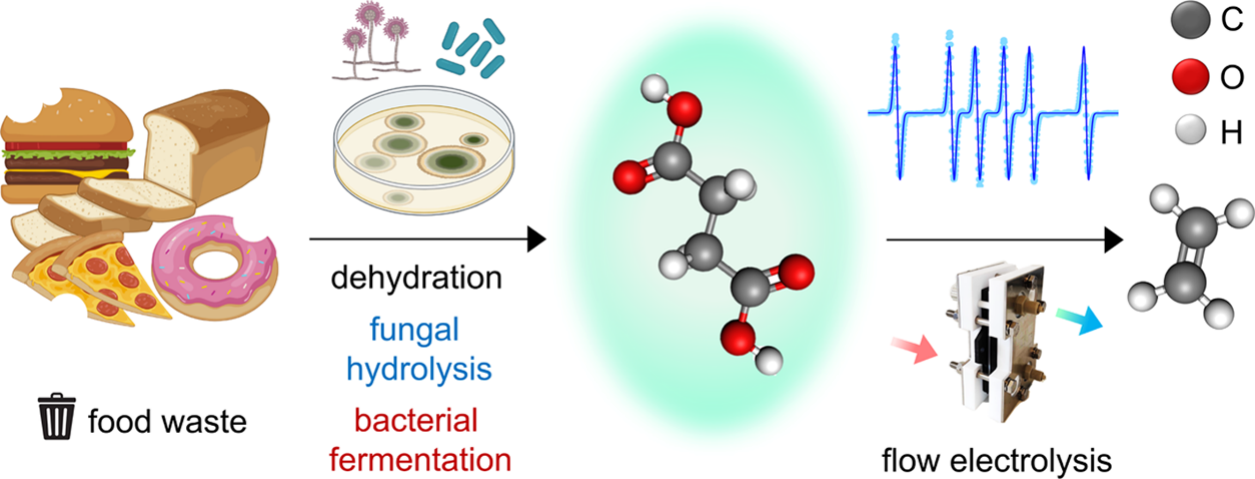

Ethylene is an important feedstock in the chemical industry, but currently requires production from fossil resources. The electrocatalytic oxidative decarboxylation of succinic acid offers in principle an environmentally friendly route to generate ethylene. Here, a detailed investigation of the role of different carbon electrode materials and characteristics revealed that a flat electrode surface and high ordering of the carbon material are conducive for the reaction. A range of electrochemical and spectroscopic approaches such as Koutecky–Levich analysis, rotating ring-disk electrode (RRDE) studies, and Tafel analysis as well as quantum chemical calculations, electron paramagnetic resonance (EPR), and *in situ* infrared (IR) spectroscopy generated further insights into the mechanism of the overall process. A distinct reaction intermediate was detected, and the decarboxylation onset potential was determined to be 2.2–2.3 V versus the reversible hydrogen electrode (RHE). Following the mechanistic studies and electrode optimization, a two-step bio-electrochemical process was established for ethylene production using succinic acid sourced from food waste. The initial step of this integrated process involves microbial hydrolysis/fermentation of food waste into aqueous solutions containing succinic acid (0.3 M; 3.75 mmol per g bakery waste). The second step is the electro-oxidation of the obtained intermediate succinic acid to ethylene using a flow setup at room temperature, with a productivity of 0.4–1 μmol ethylene cm_electrode_^–2^ h^–1^. This approach provides an alternative strategy to produce ethylene from food waste under ambient conditions using renewable energy.

## Introduction

Electrochemical processes driven by renewable energy sources hold promise in the chemical industry to reduce fossil fuel dependence and associated CO_2_ emissions.^1−3^ Alternative sustainable resources for fuel and chemical production can also contribute to attaining a net-zero carbon economy. Biomass and waste substrates (food waste, plastics, or mixed waste streams) are cheap and ubiquitous, making them an attractive alternative source to produce platform chemicals.^4−6^ Carboxylic acids are important intermediates in the upgrading of biomass and waste substrates to higher-value chemicals, as they can be obtained efficiently via various pathways.^7,8^ The chemical conversion of carboxylic acids using electrochemistry has been investigated intensively since the discovery of the Kolbe reaction, which is the oxidative dimerization of carboxylic acids to form hydrocarbons.^9−11^

Several approaches are known to convert carboxylic acids (e.g., levulinic acid, hexanoic acid) into longer-chain aliphatic compounds (fuels) or high-value organic chemicals,^3,4,12−14^ but the carboxylic acids need to be produced and purified before electrochemical conversion. Electro-biorefinery concepts combine biological and electrochemical processes to facilitate the conversion of complex renewable feedstocks into high-value chemicals.^8,12,15^ Long-chain alkanes, such as decane, obtained from the Kolbe reaction, are suitable transportation fuels, but they are not ideal substrates for the chemical industry, due to their lack of chemical functional groups.^16^ Therefore, other non-Kolbe products, such as alkenes are more desirable. Among others, ethylene, with a yearly production of 170 million tons, serves as a key intermediate owing to its high industrial demand and can be further processed into a variety of different chemicals through established routes, such as ethylene oxide, styrene, or vinylchloride.^17−19^ The electrochemical conversion of carboxylic acids into alkenes has been described as a side reaction in the Kolbe process, but alkenes have rarely been the target product in these approaches.^20−23^ Recently, the conversion of dicarboxylic acids into alkenes has been reported,^24,25^ but little is known about the mechanism of this reaction and the factors influencing the overall process. A deeper insight is necessary to enable the efficient and selective production of alkenes.

In this work, we demonstrate a combined bio-electrochemical process that allows the conversion of food waste into ethylene, with succinic acid as the central intermediate. The first biocatalytic step utilizes solid-state and aqueous-phase fermentation for the conversion of food waste into succinic acid, whereas the subsequent electrocatalytic step transforms the succinic acid intermediate to ethylene. This approach establishes an alternative and renewable pathway for the generation of ethylene from waste substrates.^19^ In this study, different approaches for material characterization and electroanalytical techniques were applied to evaluate the structure–activity relationships for the oxidative decarboxylation of succinic acid on carbon electrode materials. A reaction mechanism was proposed for this decarboxylation reaction using quantum chemical calculations, supported by electron paramagnetic resonance (EPR) and *in situ* infrared (IR) spectroscopy.

## Results and Discussion

### Analysis of Carbon Materials

**Figure 1:**
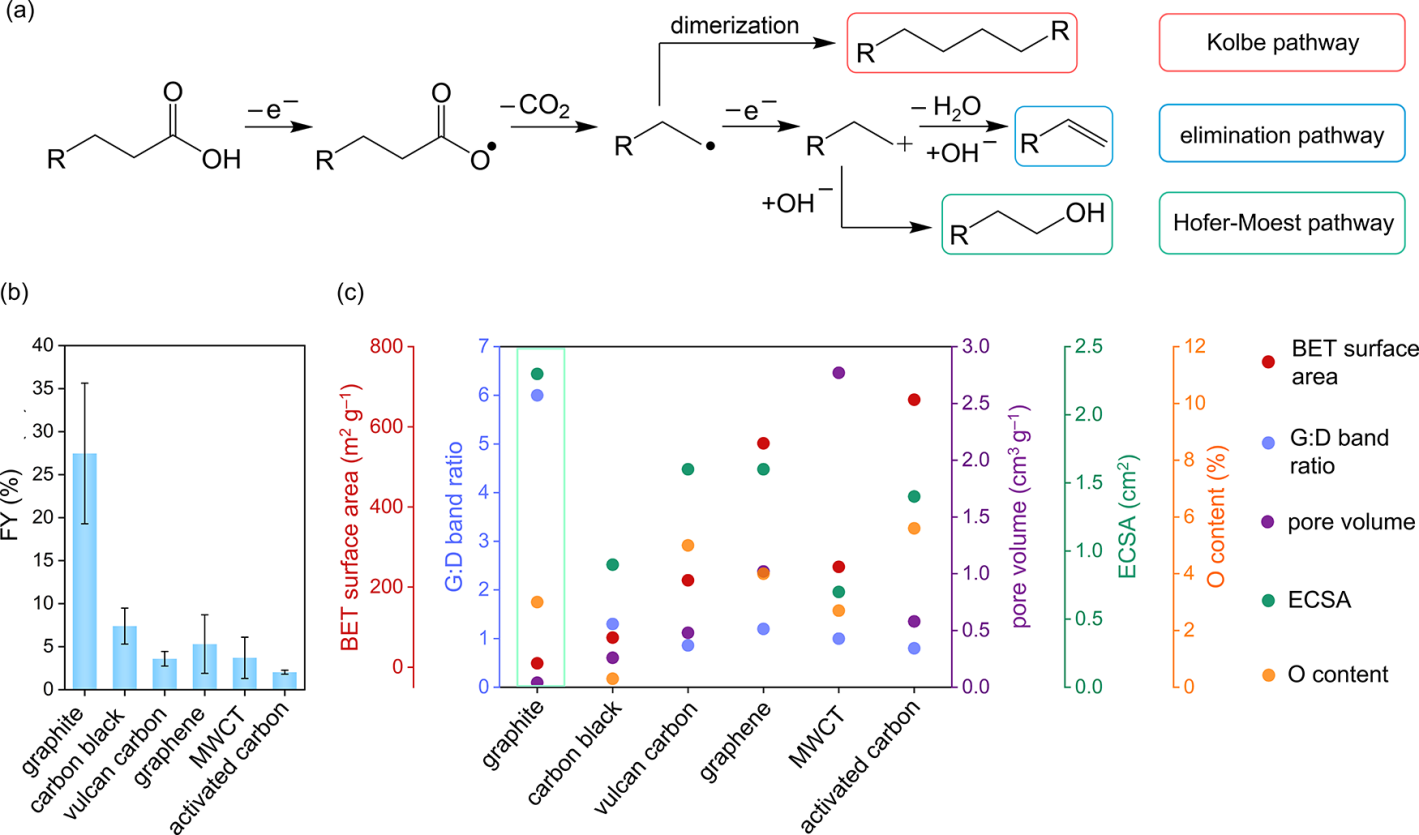
(a) Possible routes for the decarboxylation of monocarboxylic acids. (b) Faradaic yields (abbreviated as FY) for ethylene production from succinic acid on different types of carbon electrodes. Conditions: RDE setup (600 rpm); reaction solution: 0.08 M succinic acid in water (adjusted to pH 10 using NaOH); applied potential: 2.8 V vs RHE for 20 min, 25 °C. (c) Comparative overview of critical physical properties (G:D band ratio, Brunauer–Emmett–Teller (BET) surface area, pore volume, electrochemical surface area (ECSA) and O content) on each type of carbon, which influences the decarboxylation reaction of succinic acid.

The nature of the electrode material has a decisive influence on the electrochemical Kolbe oxidation of organic acids, which undergo a decarboxylation step and form an intermediate radical that dimerizes to form hydrocarbon products. Noble metals such as Pt are generally preferred as they result in a high yield of the dimerization product (Figure 1a).^4^ Electrode materials such as carbon favor the formation of carbocation intermediates, yielding a variety of products such as alcohols (via nucleophilic attack of H_2_O or ^–^OH in an aqueous medium; Hofer–Moest pathway) or alkenes (via β-elimination) as illustrated in Figure 1a.^3,26^ We therefore carried out preliminary electrochemical studies for succinic acid conversion using both Pt and carbon electrodes (counter and reference electrodes were Pt foil and Ag/AgCl_sat,KCl_, respectively; potentials were calculated and are reported with respect to the reversible hydrogen electrode (RHE) scale) by applying a potential of 2.8 V vs RHE in aqueous succinic acid (0.08 M) for 20 min at pH 10 (a high pH is conducive for the overall succinic acid conversion^25^). As anticipated from previous studies, the Pt electrodes (geometric area: 2 cm^2^) resulted in overoxidation of the substrate to CO_2_ and did not yield ethylene,^25^ whereas carbon paper and glassy carbon electrodes (geometric areas: 2 and 0.79 cm^2^, respectively) produced ethylene (detected using gas chromatography) with a faradaic yield (FY) of 29 ± 2 and 26 ± 2%, respectively. Studies with ^13^C-labeled (on C2 and C3 positions) succinic acid showed the expected doublet originating from the J coupling between the proton (^1^H) and the carbon (^13^C) of ethylene in the^1^H NMR spectra and thereby confirmed that ethylene is derived from the succinic acid (Figure S1). As shown previously, the remaining charge is mainly used for electrocatalytic water oxidation.^25^

To gain a better understanding of the influence that the electrode material properties exert on the electrochemical reaction, a diverse range of carbon materials such as graphite, carbon black (acetylene derived), Vulcan XC72 (vulcan carbon), graphene, multiwalled carbon nanotubes (MWCT), and activated carbon (Norit SA3) were investigated for their activity toward the conversion of succinic acid to ethylene. To investigate the influence of various material characteristics, the selected materials include different surface areas, porosities, and surface chemistries.

For this purpose, the different carbon materials were dispersed in ethanol (20 mg mL^–1^, 75 μL of 2 wt % Nafion117 solution mL^–1^ ethanol) and drop-cast on a gold rotating disk electrode (Au-RDE, diameter 1 mm). Only the drop-cast carbon contributes to the decarboxylation reaction as gold is inactive for succinic acid decarboxylation under the applied potentials.

The reaction solution consisted of aqueous succinic acid (0.08 M) as the model substrate, set to pH 10 with NaOH, and chronoamperometry was carried out for 20 min at 2.8 V vs RHE with a rotation speed of 600 rpm at the rotating disk electrode (RDE) (see the Experimental Section for details). The ethylene yield after the reaction was determined from the headspace using gas chromatography (GC). The FYs of ethylene for the different carbon materials after the electrochemical experiments are shown in Figure 1b and Table 1. Graphite (7–11 μm particle size) displays the highest FY for ethylene production (27.5 ± 8.1%), followed by carbon black (7.4 ± 2.1%), graphene (5.3 ± 3.4%), MWCT (3.7 ± 2.4%), vulcan carbon (3.6 ± 0.8%), and activated carbon (2.0 ± 0.2%). Furthermore, to compare the succinic acid decarboxylation with the classic Kolbe reaction, propanoic acid (a monocarboxylic acid) was also investigated as a substrate under the same conditions.^24^ Similar to the reactions with succinic acid, the electrochemical experiments showed graphite to be the most active material with a FY (for ethylene) of 27.4 ± 2.4% using propanoic acid as a substrate (Table 1).

**Table 1:** FYs using various carbon electrodes for the oxidation of succinic or propanoic acid to ethylene (0.08 M acid in H_2_O; carbon material dispersed in ethanol, 20 mg mL^–1^ with 75 μL of 2 wt % Nafion117 solution mL^–1^ ethanol and drop-cast on 1 mm diameter Au-RDE; 600 rpm, 25 °C, 20 min). Conditions: pH 10, applied potential: 2.8 V vs RHE.

	substrate: succinic acid		substrate: propanoic acid	
material	FY of ethylene (%)	ethylene yield (μmol)	FY of ethylene (%)	ethylene yield (μmol)
graphite	27.5 ± 8.1	1.09 ± 0.41	27.4 ± 2.4	1.83 ± 0.24
carbon black	7.4 ± 2.1	0.05 ± 0.03	18.9 ± 1.1	0.50 ± 0.002
vulcan carbon	3.6 ± 0.8	0.06 ± 0.04	10.2 ± 2.9	0.29 ± 0.03
graphene	5.3 ± 3.4	0.12 ± 0.02	4.8 ± 1.0	0.11 ± 0.04
MWCT	3.7 ± 2.4	0.04 ± 0.01	3.4 ± 0.9	0.07 ± 0.02
activated carbon	2.0 ± 0.2	0.02 ± 0.001	3.6 ± 2.0	0.09 ± 0.05

The carbon materials were analyzed using various analytical techniques to determine the relevant factors contributing to the activity. The powder X-ray diffraction (PXRD) patterns (Figure S2) of graphite showed a dominant peak at 2θ = 27° indicating crystallinity, whereas that of carbon black and graphene showed small broader peaks in the same region, indicating a lower degree of crystallinity. The other carbon materials (vulcan carbon, MWCT, and activated carbon) did not exhibit any significant peaks, indicating a low share of crystalline graphitic structure. Raman spectroscopy is a suitable complementary method to assess the ordering of the carbon structure and to determine structural imperfections. Two distinct Raman bands are observed in the carbon materials: the G-band at 1570 cm^–1^, which represents the ordered graphite-like structure, and the D-band at 1370 cm^–1^, which indicates the degree of disorder in the carbon structure. Moreover, an additional feature corresponding to disordering appears as the small D′ band at 1630 cm^–1^.^27^ The ratio of the areas of the G to D bands (G:D ratio) is a qualitative measure for the ordering of the carbon structure. Among the different carbon materials tested for our reaction (the individual Raman spectra are shown in Figure S3), graphite exhibited the highest G:D ratio of 6 (Figure 1c; Table S2), followed by carbon black, graphene, MWCT, vulcan carbon, and activated carbon (with values of 1.3, 1.2, 1, 0.9, and 0.8, respectively; Figure 1c; Table S2).

In addition to the degree of order in a structure, the surface area and chemical composition can also play a crucial role in the (electro)catalytic behavior of materials. Therefore, N_2_-physisorption measurements were conducted to determine the Brunauer–Emmett–Teller (BET) specific surface area and pore volume of the different carbon materials (Figure 1c; Table S2). Graphite showed the lowest BET surface area (9.6 m^2^ g^–1^) and negligible pore volume (0.04 cm^3^ g^–1^). Activated carbon had the highest BET surface area of 667 m^2^ g^–1^, whereas MWCT exhibited the highest pore volume (2.8 cm^3^ g^–1^) with its hollow nanosized tubular structure. Although the BET surface area provides a physical notion of the catalyst material, for electrochemical reactions, the electrochemical surface area (ECSA) provides more relevant information about the active sites participating in the process. Among the different types of carbon catalysts used for our purpose, graphite showed the highest ECSA of 2.3 cm^2^, suggesting a high number of electrochemically accessible sites (Figure 1c; Table S2; Figure S4).

The chemical composition of the electrode surface was determined using X-ray photoelectron spectroscopy (XPS) (Figure S5). All materials showed a single main carbon peak at 284 eV (attributed to the sp^2^ hybridized carbon) and a smaller peak at ∼286 eV corresponding to C–O groups (Figure S5). No sp^3^ carbon was found in any of the carbon materials used in our study. The oxygen content on the surface of the carbon materials was estimated and found to range from 0.3% (for carbon black) and 5.6% (for activated carbon) as shown in Figure 1c.

Although correlating the physical properties of carbon materials to their catalytic activity is challenging, our analyses provide certain insights into the overall process. Higher crystallinity and ordering (less defects) of the structure (as determined using PXRD and Raman spectroscopy) appear to be beneficial for the reaction, with graphite displaying the highest in these parameters and showing the best electrochemical activity. Carbon black, which exhibits the second highest G:D ratio (1.3) after graphite (6.0), was found to show the second highest activity. In addition, our analyses also suggest that flat surfaces are preferred over high pore volumes. This is evident from our initial electrochemical experiments with glassy carbon (negligible porosity),^28^ which also showed a high FY towards ethylene formation (26 ± 2%), despite not having a particularly ordered structure.^28^ The detrimental effect of porous materials can be explained in two possible ways. First, during the electrochemical process, it is possible for the highly reactive intermediates to undergo side reactions if their diffusion to and from the electrode surface is slowed down in the presence of a porous structure; second, a large pore volume may not translate into substrate accessibility, as materials with large pore volumes (such as MWCT) show a comparatively low ECSA value. The surface oxygen content in the carbon materials does not seem to have a significant influence on the catalytic activity.

### Electrochemical Analysis of the Decarboxylation Reaction

Due to its higher catalytic activity, graphite was further investigated as the electrode material for succinic acid to ethylene conversion using electrochemical analytical techniques to obtain a deeper understanding of the overall catalytic process. Koutecky–Levich analysis was performed to gain insights into the reaction proceeding at the electrode (see the Supporting Information for details). Therefore, the rotation speed (600–4000 rpm) of the RDE was varied at different potentials (2.1–2.8 V vs RHE). For these tests, the graphite suspension (in ethanol; 20 mg mL^–1^, 75 μL of 2 wt % Nafion solution mL^–1^ ethanol) was drop-cast on Au-RDE, with Pt foil and Ag/AgCl as the counter and reference electrodes, respectively (see the Experimental Section for details, potentials reported vs RHE). The reaction solution consisted of aqueous succinic acid (or propanoic acid; 0.01 M) at pH 10.

**Figure 2:**
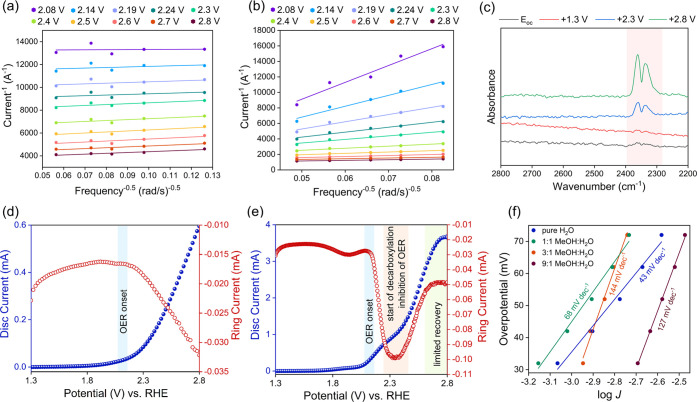
(a, b) Koutecky–Levich plots (5 μL of suspension drop-cast on Au-RDE; the suspension contains 20 mg graphite mL^–1^ ethanol and 75 μL of 2 wt % Nafion solution mL^–1^ ethanol) in pure water (a) and 0.01 M succinic set to pH 10 with NaOH (b). The potentials indicated are vs RHE. (c) *In situ* IR measurements at different applied potentials (vs RHE). (d, e) RRDE experiments with a glassy carbon disk (5 mm diameter, voltage screen 1.3–2.8 V vs RHE) and Pt ring (constant potential: 0.1 V vs RHE) with 0.01 M (d) and 0.24 M (e) aqueous succinic acid set to pH 10 with NaOH (N_2_ purged for 20 min, rotation speed: 600 rpm). (f) Tafel plots (0.01 M succinic acid in different MeOH:H_2_O solutions, carbon paper electrode (2 cm^2^), Pt foil counter and Ag/AgCl reference, *V*_0_ = 1.28 V to calculate the overpotential). The experiments were conducted at room temperature.

In the absence of the succinic acid substrate (with pure H_2_O, set to pH 10 with NaOH), Koutecky–Levich plots showed similar slopes at all applied potentials (2.1–2.8 V vs RHE; Figure 2a). However, when succinic acid was present, the change in slopes of the Koutecky–Levich plots was evident when moving from higher to lower potentials, with a rapid increase in slopes at lower potentials as observed in Figure 2b. The slopes of the Koutecky–Levich plots are shown in Table S3. A similar trend was also observed when propanoic acid was used as a substrate instead of succinic acid (Figure S6). The variation of the slope of the Koutecky–Levich plots in the presence of succinic acid (or propanoic acid) indicates a change in the reaction mechanism when going from lower to higher potentials. The point at which the slope variation starts, corresponds to the onset of the decarboxylation reaction (lying between ca. 2.2 and 2.3 V vs RHE). Water oxidation dominates at lower potentials (characterized by higher slopes; see below). These results indicate that the decarboxylation reaction (with an onset between ca. 2.2 and 2.4 V vs RHE) and water oxidation occur in parallel, with the former gaining more prominence at higher potentials.

The potential range for the onset of decarboxylation was also confirmed using *in situ* IR spectroscopy shown in Figure 2c. For the *in situ* IR studies, thin films of graphite powder were deposited on Si prisms by spin coating. The prisms were mounted in custom-made cells and covered with 0.01 M aqueous succinic acid solution (set to pH 10 with NaOH). The IR studies showed that CO_2_ evolution (from decarboxylation) commences from ∼2.3 V vs RHE, whereas no CO_2_ bands were detected at lower potentials, or if no succinic acid was present (Figure S7). This also indicates that the overoxidation of the carbon electrode to CO_2_ is negligible under the applied reaction conditions.

The concurrent decarboxylation and water oxidation reactions were also studied using rotating ring-disk electrodes (RRDE; glassy carbon disk with 5 mm diameter and Pt ring). In the RRDE setup, an anodic potential sweep was performed (1.3–2.8 V vs RHE) to drive the decarboxylation reaction, with the oxygen evolution reaction occurring simultaneously, as determined above (see the Experimental Section for details).^25^ The Pt ring electrode, where a constant cathodic potential (0.1 V vs RHE) is applied, reduces the oxygen evolved at the adjacent disk electrode (oxygen reduction reaction (ORR)) resulting in an increase in the negative (cathodic) current. The onset of the decarboxylation reaction at higher potentials should partially suppress the oxygen evolution reaction. The RRDE tests were performed using three different concentrations of succinic acid (0.01, 0.08, and 0.24 M). Figures 2d,e and S8 show that the disk current (blue) appears to be similar in all of the cases with the current rising sharply between 2.2 and 2.3 V vs RHE. Moreover, the onset of water oxidation at the disk coincided with the onset of the ORR, which is evident from the increase in the ring current (red). Very low succinic acid concentrations (0.01 M) did not affect the continuous production of oxygen at the carbon disk (Figure 2d). With an increase in the succinic acid concentration (0.08 or 0.24 M), the ring current decreased and plateaued when the carbon disk electrode reached a potential range of 2.2–2.3 V vs RHE (Figure 2e and Figure S8). The decrease is more pronounced at the higher succinic acid concentration (0.24 M), where limited recovery of the ring current was observed (Figure 2e) at higher potentials (>2.5 V vs RHE). These observations indicate that the oxygen production at the carbon disk electrode is partially suppressed by the decarboxylation reaction. The RRDE experiments with propanoic acid as the substrate showed similar trends (Figure S9). These experiments confirm that the decarboxylation and water oxidation occur as parallel reactions on the carbon electrodes and the onset of decarboxylation aids the suppression of oxygen evolution, particularly at high concentrations of the acid substrate. Furthermore, the onset of ethylene evolution lies between 2.2 and 2.3 V vs RHE, in agreement with the Koutecky–Levich and *in situ* IR spectroscopic analyses.

We have observed in previous studies that the presence of methanol (MeOH) benefits the decarboxylation reaction and increases the FY of ethylene.^25^ To investigate this further, Tafel analysis was conducted, whereby the electrochemical experiments were performed using carbon paper (graphitic) electrodes (2 cm^2^) (counter electrode: Pt, 2 cm^2^; reference electrode: Ag/AgCl) and the media consisted of different ratios of MeOH:H_2_O (1:1, 3:1, and 9:1) with aqueous succinic acid (0.01 M) at pH 10. The Tafel slopes increased for high MeOH concentrations in the presence of succinic acid (>100 mV dec^–1^ for 3:1 and 9:1 MeOH:H_2_O), indicating slower kinetics between the electrode–solution interface (Figure 2f). In the case of low MeOH concentrations (1:1) or purely aqueous succinic acid solutions, the Tafel slopes were lower (68 and 43 mV dec^–1^, respectively). It is important to note here that the parallel reactions of decarboxylation and water oxidation have a considerable effect on the Tafel slopes. The water oxidation reaction, prevalent at high aqueous concentrations, becomes the dominant factor resulting in low Tafel slopes or faster kinetics. At high MeOH concentrations, however, the water oxidation is suppressed, and the decarboxylation becomes more pronounced. This results in higher Tafel slopes as the decarboxylation reaction is kinetically more demanding than the water oxidation reaction.^29^ This further supports the experimental observations where the presence of MeOH as an auxiliary solvent diminishes OER, favoring decarboxylation of succinic acid to finally enable ethylene formation with higher FYs. The possible overoxidation of MeOH was probed by performing chronoamperometric studies with a blank 9:1 MeOH:H_2_O solution (set to pH 10 with NaOH; 2.8 V vs RHE for 2 h). However, no CO_2_ could be detected in the gas phase, indicating that the oxidation of MeOH to CO_2_ is negligible under the applied conditions. Similar blank experiments under aqueous conditions (set to pH 10 with NaOH) also resulted in no CO_2_ production, suggesting that the carbon electrode material is stable under the applied electrochemical conditions.

### Quantum Chemical Study and EPR Spectroscopy

**Figure 3:**
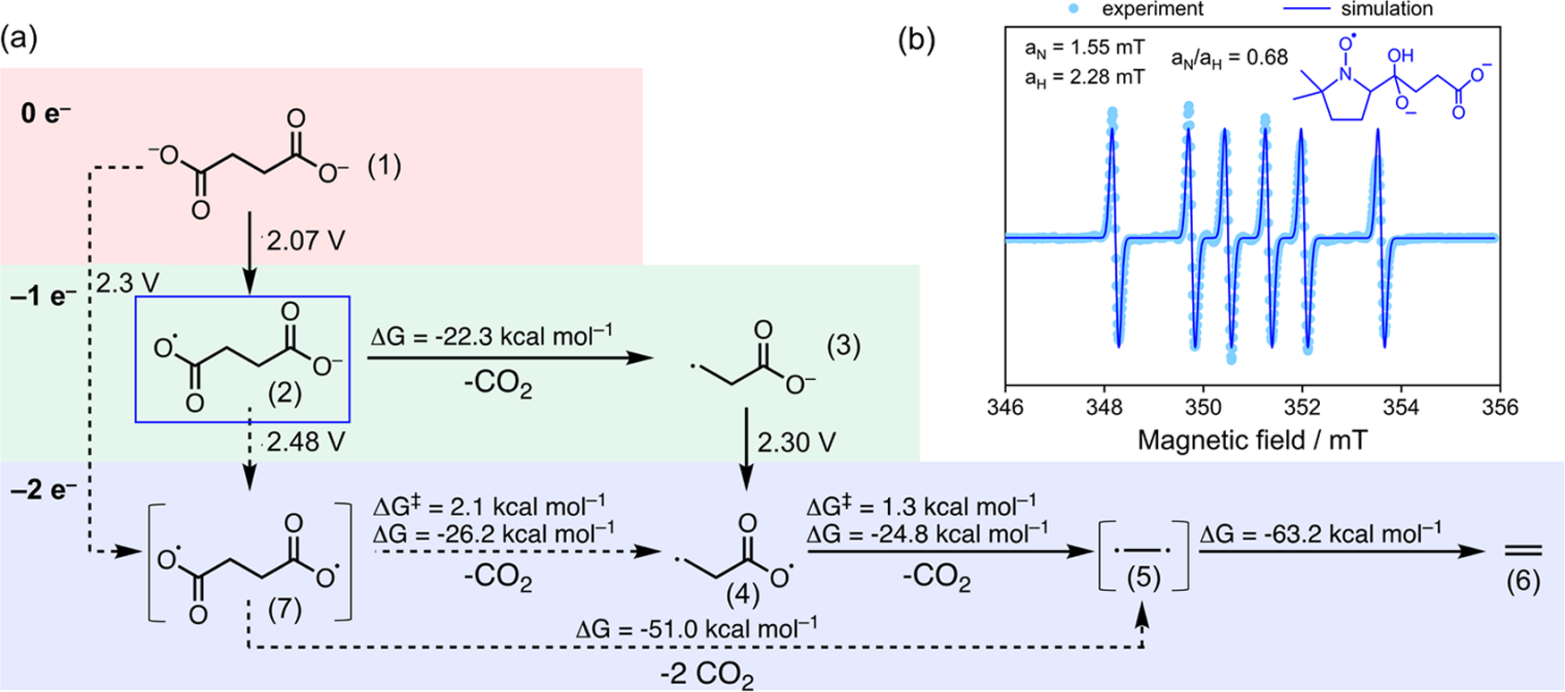
(a) Quantum chemical calculation of the reaction network (geometry optimization and vibrational analysis were performed with a def2-TZVP basis set using the M06–2X functional). The potentials indicated are vs RHE. (b) Spin trapping of 90 mM succinic acid in 150 mM NaOH pH 10 with 50 mM 5,5-dimethyl-1-pyrroline *N*-oxide (DMPO). The adduct is trapped at 20 min reaction time (light blue dots) and the corresponding simulation (dark blue line) suggests the formation of intermediate (2) in (a). Inset: chemical structure of the DMPO + (2) adduct. Simulation parameters *a*_N_ = 1.55 mT, *a*_H_ = 2.28 mT, *g* = 2.0084.

Previous studies investigating the decarboxylation of monoacids (such as propanoic acid) suggested that the reaction proceeds by either the Kolbe radical pathway or via a carbocation intermediate (Figure 1a).^10,23^ The exact mechanism for the decarboxylation has not been elucidated for succinic acid so far, but a radical mechanism has been postulated for diacids.^24^ Thus, mechanistic studies for succinic acid decarboxylation yielding ethylene were performed using density functional theory calculations (see the Experimental Section and Supporting Information for details). The reaction network in Figure 3a depicts several plausible pathways for product formation. The sequential reaction pathways start with the oxidation of the deprotonated succinic acid (1) to the carboxylate radical (2). The decarboxylation of this carboxylate radical is exergonic and the product, alkyl radical (3), can undergo a second oxidation step, to the unstable diradical (4). Intermediate (4) would be decarboxylated again and, via the hypothetical intermediate (5), spontaneously form ethylene (6) as the product. Alternatively, the succinic acid could potentially also form a diradical (7) that undergoes a double decarboxylation to form ethylene via the hypothetical intermediate (5). The calculated potentials for the oxidative steps (2.07–2.27 V vs RHE) fit well to the observed onset potentials of ethylene formation in the previous experiments (2.2–2.3 V vs RHE).

Spin trapping EPR experiments were performed to gather experimental information on the reaction mechanism. This technique allows us to observe short-lived radical species by converting them into more stable ones. For our purpose, 5,5-dimethyl-1-pyrroline-*N*-oxide (DMPO) was used as the spin-trapping agent for adduct formation during EPR measurements.

The CW–EPR spectrum revealed a distinct signal pattern between 347 and 355 mT, which could be simulated as resulting from the hyperfine coupling of the unpaired electron spin with one ^14^N nucleus (*a*_N_ = 1.55 mT) and one ^1^H nucleus (*a*_H_ = 2.28 mT) (Figure 3b). The corresponding *a*_N_/*a*_H_ value of 0.68 is consistent with an adduct originating from trapping a carbon-centered alkyl radical.^30^ This conclusion is also supported by the individual values of *a*_N_ and *a*_H_.^31^ Similar parameters, characterized by the large hyperfine splitting constant for the β hydrogen, have been reported for DMPO spin adducts of carbon-centered 1-hydroxyalkyl radicals, whose EPR spectra show a characteristic asymmetry of the nitrogen triplets associated with the presence of diastereoisomeric intramolecularly hydrogen-bonded adducts; this latter asymmetry is observed in our experimental spectrum as well.^32−34^ The presence of CO_2_ radical adducts could be excluded by comparison with previous studies, as well as the potential side reaction products from the electrolyte (Figure S10).^35^ Furthermore, control experiments with succinic acid ^13^C-labeled on the C_2_ and C_3_ positions yielded the exact same CW–EPR spectrum as with unlabeled succinic acid and, based on the absence of the expected additional splitting due to the hyperfine coupling with the ^13^C nucleus, a radical on positions C_2_ and C_3_ can be excluded (Figure S11). In conjunction with the reaction network presented in Figure 3a, the corresponding trapped species is identified as the carbon-centered anionic radical intermediate (2) (Figure 3b), suggesting the stability of such a species in accordance with previous studies.^36,37^

Besides this intermediate, no other EPR signals were detected. These findings support our claim that the conversion of succinic acid into ethylene proceeds through the sequential decarboxylation route via the monoalkyl radical (3), whereby the second oxidation step, occurring via the hypothetical intermediates (4) and (5), is extremely fast and results in the formation of ethylene. Because of the very short reported lifetime of carboxyl radicals,^38^ potentially preventing the detection of these species through spin trapping, the direct 2e^–^ oxidation via the diradical species (7)^24^ cannot be yet ruled out completely albeit our experiments suggest that it is unlikely.

### Bio-Electrocatalytic Conversion of Food Waste to Ethylene in Flow

**Figure 4:**
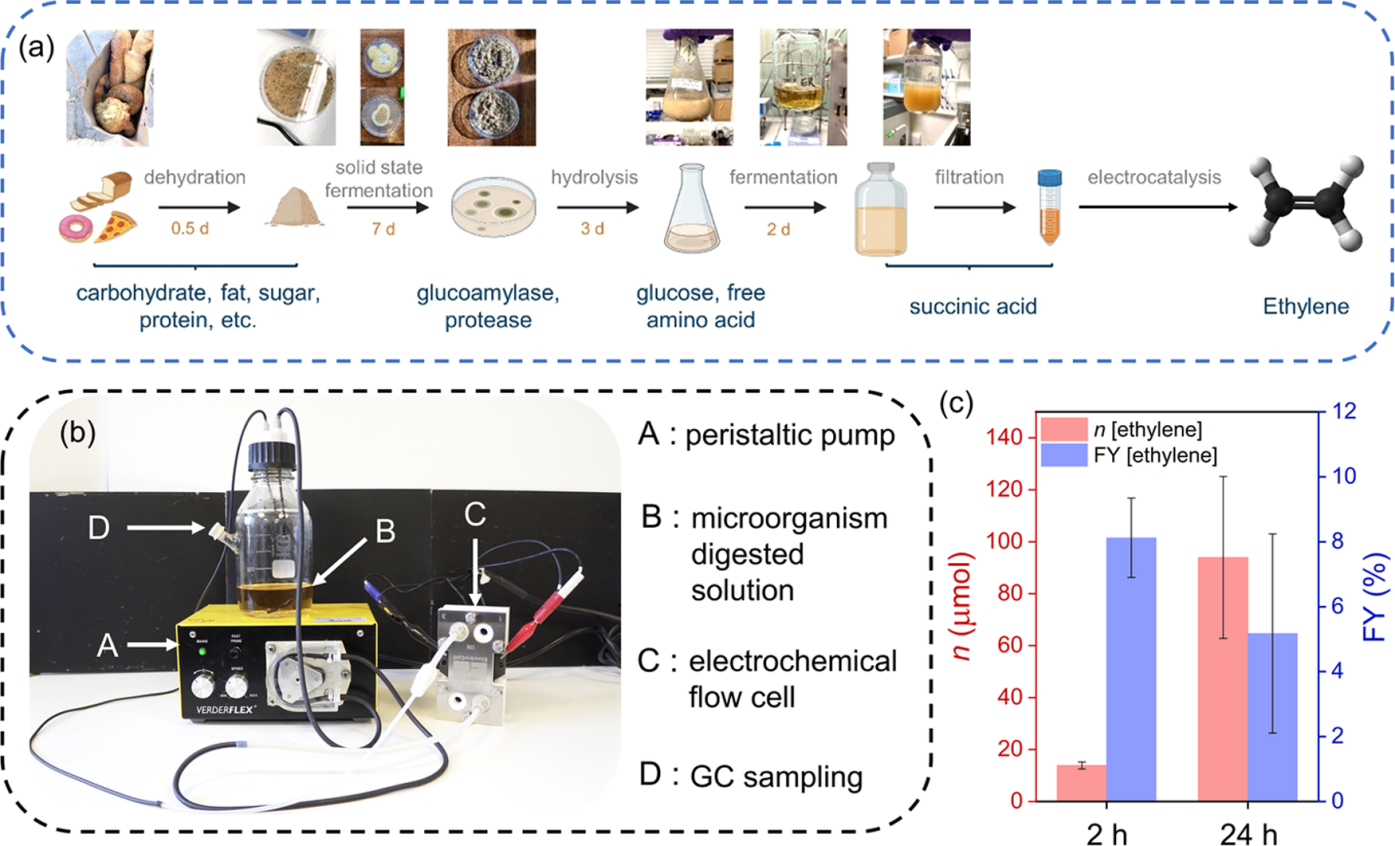
(a) Flow scheme bio-electrocatalytic process. (b) Digital photograph of the electrochemical flow setup with labeled components. (c) Plots showing the ethylene production and FYs from the microorganism-digested food waste solution using the flow electrochemical setup for 24 h. Conditions: *E*_cell_: 3 V, pH 6, room temperature.

A detailed investigation of the electrochemical conversion of succinic acid to ethylene through physical analysis of the materials, electroanalytical techniques, density functional theory calculations, and EPR provided critical insights into the properties of electrode materials conducive for the reaction and the mechanism of the overall decarboxylation process. We next aim to demonstrate the wider importance of the reaction in terms of practical utility where ethylene can be generated from waste feedstocks via a succinic acid intermediate. For this purpose, we designed and developed a combined bio-electrocatalytic approach (Figure 4).

The first step in the process involves biocatalytic conversion of food waste (waste bread) into succinic acid (Figure 4a; see the Experimental Section for details).^39,40^ The food waste suspension is treated by a combination of microorganisms to obtain a clear yellow fermentation broth with pH ∼6 and containing ∼0.3 M succinic acid detected by high-performance liquid chromatography (HPLC) analysis. (Conditions: 7 days solid-state food waste fermentation at 30 °C with *Aspergillus awamori* and *Aspergillus oryzae* followed by enzymatic hydrolysis: 20 g of solid-state fermented food waste, 150 mL of H_2_O, incubated for 3 days at 55 °C; followed by filtration and centrifugation; *Actinobacillus succinogenes* was added to this solution and fermented for 2 days at 37 °C, filtrated and centrifuged to obtain final solution). The microorganism-digested solution (containing succinic acid) was then used as the substrate for the two-electrode electrolysis experiments with graphitic carbon paper (2 cm^2^) as the anode (and Pt cathode) with an applied voltage of 3 V in a 20 mL single-compartment electrochemical cell. The native pH of 6 was kept unchanged as the addition of NaOH caused precipitation of unknown byproducts from the fermentation process. Furthermore, the addition of NaOH for the pH adjustment would be disadvantageous for the economic feasibility of the process. The single-compartment, two-electrode electrolysis setup was chosen as it is less cumbersome and hence, more cost-effective and facile to utilize from the perspective of real-world applications. While ethylene will be formed at the anode, the main cathodic reaction is hydrogen evolution. After 2 h of a controlled potential electrolysis (CPE) experiment, the headspace was analyzed using GC for the detection and quantification of the ethylene and H_2_ formed (CV scans and CPE traces are shown in Figure S12). The FY for ethylene formation from the microorganism-digested solution was ∼10 ± 5% (21.3 ± 13.5 μmol after 2 h) (Table S4).

We also employed an electrochemical flow reactor (electrolyzed with an electrode area of 10 cm^2^ in a single-compartment configuration; see Figures 4b, S13, and Movie S1) to enhance practicality for the continuous conversion of larger amounts of the fermentation broth. Solid graphite electrodes were used as the anode and cathode, as graphite showed the best activity for the reaction from our previous experiments. A glass reservoir contained the reaction solution (microorganism-digested solution or pure succinic acid solution) which was continuously pumped through the flow reactor during the CPE experiments at 3 V for 24 h (Figure S12). The gaseous reaction products accumulated in the reservoir and were analyzed using GC. After 24 h of electrochemical flow experiments with the fermentation broth, 94 ± 31 μmol of ethylene was obtained with a FY of ∼5 ± 3% (Figures 4c, S12c,d, Table S4). Besides ethylene, CO_2_ (6 ± 0.3 μmol) and CO (<0.5 μmol) were also detected as side products from the decarboxylation reaction. The other major gaseous product was H_2_ from the cathodic counter reaction (1257 ± 466 μmol after 24 h; FY_H2_ = 63 ± 5%). The side products in the liquid phase, such as adipic acid,^25^ were not quantified due to the complex composition of the fermentation broth, which masked the appearance of additional peaks in the HPLC chromatogram. These side products can potentially also engage in the electrochemical reactions, lowering the FYs. Nevertheless, the two-step bio-electrocatalytic flow experiments demonstrated the conversion of food waste into ethylene via succinic acid as an intermediate product.

### Comparison with Other Approaches

Other types of bio-electrochemical processes for the generation of chemical products have previously been reported with different process design and target products. The process reported in this study starts with the biocatalytic transformation of complex food waste into the succinic acid intermediate. This is followed by an electrocatalytic step to anodically convert the succinic acid into ethylene. Other approaches commonly start with an electrocatalytic step followed by biocatalysis. Electrolysis allows for the generation of gaseous intermediates such as H_2_ from water splitting or CO from CO_2_ reduction.^15,41^ For example, syngas (CO + H_2_) production has been reported with a CO_2_ electrolyzer setup, which can then be transferred into a fermenter reactor where *Clostridium* bacteria species convert it into alcohols such as butanol and/or hexanol.^15^ The final product is an aqueous solution containing 14–28 mmol alcohol L^–1^ (depending on the fermentation time). For a combined CO_2_ reduction + fermentation process, the alcohol yield was stated to be approximately 0.5 mol alcohol per kWh utilized in the CO_2_ electrolyzer.^15^ Using the unoptimized parameters for the process described in our report, the ethylene yield would be ∼0.25 mol kWh^–1^, and therefore in a similar range as the previously reported processes (see equation 2 in the Supporting Information for details). However, the CO_2_-reduction + fermentation process operates with the oxygen evolution reaction as the counter reaction in the electrochemical step, while in the present study the balancing reaction is the hydrogen evolution reaction. This allows the generation of two valuable products namely, ethylene and hydrogen, whereas in the previously reported process a mixture of alcohols is the sole product. Another factor is product isolation. It is suggested that the alcohols can be isolated by extraction with oleylalcohol and subsequent distillation. The present ethylene product also has to be separated from the H_2_ and CO_2_ byproducts. However, the energy requirement for distilling hexanol from oleylalcohol is 2.2 MJ kg^–1^ alcohol, whereas gas purification via membrane separation requires only ca. 0.8–0.9 MJ kg^–1^ ethylene.^15,42−44^ Furthermore, ethylene can be purified in one process step, whereas the alcohols require an extraction and a distillation step. As the present process yields gaseous ethylene, this is a significant advantage regarding downstream product purification compared to other processes yielding liquid products.

Ethylene can also be obtained by direct electrochemical reduction of CO_2_ with a FY > 60%, reaching current densities up to 1 A cm^–2^.^45−47^ If the electricity is provided from renewable sources, this can be considered another sustainable possibility for ethylene production. However, the CO_2_ to ethylene reduction reaction requires 12 electrons, whereas the oxidative decarboxylation of succinic acid to ethylene requires only 2 electrons. Although the presented bio-electrocatalytic process still requires further optimization, similar ethylene yields per Coulomb can be achieved as the lower FY of the decarboxylation reaction is compensated by the lower number of required electrons. Efficient implementation of direct CO_2_ reduction also requires concentrated CO_2_ sources, from large CO_2_ emitters, limiting its applicability. Additionally, the balancing half-reaction for the direct CO_2_ to ethylene reduction is usually the oxygen evolution reaction. As succinic acid decarboxylation is an anodic reaction, it can either be balanced with the hydrogen evolution reaction at the cathode or CO_2_ reduction at the cathode, yielding two valuable products in a single electrolysis reaction.

Currently, ethylene is produced industrially in petrochemical steam cracking processes, utilizing either naphtha or ethane as feedstock. These processes operate at high temperatures between 750 and 950 °C, which can only be performed efficiently on a large and centralized petrochemical refinery, to benefit from the economy of scale. Alternative electrochemical or bio-electrochemical processes could potentially operate in a smaller, more decentralized manner, providing novel business models for the chemical industry. The cracking processes require an energy input of 15–40 MJ kg^–1^ ethylene and emit 1–2 kg CO_2_ kg^–1^ ethylene.^48−50^

Energy input and CO_2_ emissions were also calculated for the reported process (see the Supporting Information for details). The energy balance for ethylene alone appears to be unfavorable, as a 138 MJ energy input is required per kg ethylene. However, it should be considered that per kg ethylene also 0.89 kg H_2_ is generated in this process. If the energy content of the formed H_2_ is utilized to drive the process, the overall energy input would be reduced to 31 MJ kg^–1^ ethylene, which is comparable to highly optimized, current state-of-the-art petrochemical processes. The petrochemical processes also rely on energy input provided solely derived from fossil fuels (due to the high temperatures required), whereas for the reported process exclusively renewable electricity can be used, which limits CO_2_ emissions.

The main source of CO_2_ emission for the reported process is the 2 mol CO_2_ per mol ethylene from the decarboxylation reaction (3.1 kg CO_2_ kg^–1^ ethylene). At the same time, avoided CO_2_ emissions can be included in the balance as the utilized food waste is not composted (which would release CO_2_) but consumed in the process.^51^ If this is considered, the process would be even net CO_2_ negative with approximately −2 kg CO_2_ kg^–1^ ethylene produced. Moreover, the CO_2_ generated via decarboxylation at the anode could also be utilized for CO_2_ reduction reactions *in situ* at the cathode to produce useful chemicals.

The simplified considerations above demonstrate that the reported process harbors potential in the future, and it can be expected that continuing optimization will further reduce the energy requirement and increase productivity, to develop it into a competitive alternative to the existing industrial processes. Importantly, the reported bio-electrochemical process does not require any fossil fuels for energy generation or fossil feedstocks as substrates.

## Conclusions

We have investigated the targeted anodic decarboxylation of succinic acid to ethylene and elucidated structure–activity relationships for the carbon-based electrocatalyst. It was found that the nature of the carbon material significantly influences the reaction outcome and well-ordered, two-dimensional electrode materials are preferred, such as flat graphite. Electrochemical experiments and *in situ* IR spectroscopic measurements support the determination of the onset potential for the decarboxylation reaction ranging between 2.2 and 2.3 V vs. RHE. Insights into the reaction network were gained using quantum chemical calculations, and the presence of a monoalkyl radical as a reaction intermediate was confirmed by EPR spectroscopy. Finally, the utility of succinic acid to ethylene decarboxylation was exemplified by demonstrating a combined bio-electrocatalytic approach converting food waste into succinic acid and subsequently into ethylene (0.4 μmol ethylene cm^–2^ h^–1^). To further develop this process, it should be integrated in real-world process chains, opening new venues for waste valorization into ethylene and hydrogen as valuable products.

## Experimental Section

### Materials

Succinic acid (>99%), propanoic acid (99.5%), sodium hydroxide (>99%), multiwalled carbon nanotubes (MWCT), and activated carbon Norit SA3 were purchased from Sigma-Aldrich. Graphene nanoplatelets, carbon black (acetylene), graphite powder (7–11 μm particle size), and Toray carbon paper (uncoated) were obtained from Alfa Aesar. All chemicals and materials were used as received without further purification.

### Electrochemical Experiments with Carbon Materials

All electrochemical experiments were conducted with an Ivium CompactStat potentiostat. 17–24 mL of the particular reaction solution (containing succinic or propanoic acid) was purged with N_2_ for 20 min before conducting the tests. A three-electrode setup consisting of a Ag/AgCl (sat. KCl; BasiMW-2030) reference (potentials were recalculated vs RHE), a platinum foil counter electrode, and a working electrode (either carbon paper with 2 cm^2^ electrode area or drop-cast carbon suspension (5 μL) on Au-RDE with an area of 0.78 mm^2^) was used for the experiments. For drop-casting, the particular carbon material was dispersed in ethanol and 2 wt % Nafion solution (concentration: 20 mg mL^–1^; 75 μL of 2 wt % Nafion solution mL^–1^ ethanol). For RRDE measurements, a glassy carbon disk (5 mm diameter) with a Pt ring was used. The reaction products were monitored by manual sampling and analyzing aliquots of the reaction vessel headspace (50 μL) by GC at the end of the reaction.

### Material Analysis

#### X-ray Powder Diffraction (XRD)

XRD was conducted on a PANalytical Empyrean Series 2 instrument using Cu Kα irradiation.

#### Raman Spectroscopy

Raman spectra were acquired with a Horiba Scientific, Labram HR Evolution, and a 473 nm laser.

#### Nitrogen Physisorption Measurements

Nitrogen physisorption was obtained using a Micromeritics TriFlex porosimeter. The pretreatment temperature for the materials was 180 °C.

#### X-ray Photoelectron Spectroscopy (XPS)

XPS was performed on a Thermo Fisher Scientific K-α+ spectrometer. Samples were analyzed using a microfocused monochromatic Al X-ray source (72 W) over an area of ∼400 μm. Data were recorded at pass energies of 150 eV for survey scans and 40 eV for high-resolution scans with 1 and 0.1 eV step sizes, respectively. Charge neutralization of the sample was achieved through a combination of both low-energy electrons and argon ions. Three well-separated areas were selected on each sample for analysis to examine any surface heterogeneity. Data analysis was performed in CasaXPS using a Shirley-type background and Scofield cross sections, with an energy dependence of −0.6.

#### Electrochemical Surface Area (ECSA) Determination

The carbon material suspension (5 μL, concentration: 20 mg mL^–1^; 75 μL of 2 wt % Nafion solution mL^–1^ ethanol) was drop-cast on the 1 mm diameter Au-RDE. Cyclic voltammetric (CV) scans between 0 and 0.5 vs Ag/AgCl (0.8 and 1.3 V vs RHE) (corresponding to the nonfaradaic region of the reaction) at different scan rates (10, 20, 40, and 80 mV s^–1^) were performed without rotation to determine the double-layer capacitance (*C*_dl_) of the particular materials. By assuming a specific capacitance (*C*_s_) of 40 μF cm^–2^ for the materials, according to previous studies.^52,53^ The ECSA can be calculated using the following equation
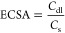


#### Tafel Plots

Succinic acid (0.01 M) in 20 mL MeOH:H_2_O solutions with various ratios (9:1, 3:1, 1:1) and only H_2_O was used for the electrochemical tests. The solution was purged with N_2_ for 20 min. Carbon paper (2 cm^2^) was used as a working electrode, Pt foil as a counter electrode (2 cm^2^), and Ag/AgCl as a reference electrode. The investigated potential range was between 2.3 and 2.8 V (vs RHE), and the potentials were corrected for the IR drop. To determine the cell resistance *R*, impedance spectra with 21 frequencies at 0.5 V were measured for each reaction solution (MeOH + H_2_O + substrate), and *R* was determined by fitting with a suitable equivalent circuit.

A similar setup was used for the blank chronoamperometric experiments under pure aqueous or MeOH:H_2_O conditions (set to pH 10 using NaOH) at 2.8 V vs RHE for 2 h under ambient conditions.

#### Electron Paramagnetic Resonance

An aqueous solution containing 90 mM succinic acid in 150 mM NaOH in the presence of 50 mM 5,5-dimethyl-1-pyrroline *N*-oxide (DMPO) as the spin trap was loaded in a one-pot electrochemical cell equipped with a three-electrode setup; the electrodes were connected to a Metrohm μAutolabIII potentiostat.

The working electrode, made of graphitic carbon paper (0.6 cm^2^ geometric area per side), was held at a constant potential of +2.8 V vs RHE (Ag/AgCl 3 M KCl, DRI-REF-2 mini-reference electrode, World Precision Instruments) using a Pt wire as the counter electrode (Scientific Glassblowing Service, University of Southampton; Pt from Goodfellow); the oxidation was performed at room temperature under bubbling with nitrogen.

The sample for CW–EPR spectroscopy was removed from the electrochemical cell 20 min after starting the experiment, loaded into a 50 μL glass micropipette (Brand BLAUBRAND intraMark), and measured immediately after.

The EPR measurements were performed at room temperature using a Bruker EMX spectrometer equipped with a Bruker premiumX microwave bridge and a Bruker ER4122SHQE high-sensitivity resonator. The spectrum was recorded as a single scan using a microwave power of 2 mW, a field modulation of 0.1 mT at 100 kHz, a conversion time of 163.84 ms (resulting in a scan time of 167.77 s), and a time constant of 40.96 ms.

The simulation of the spectrum was performed using the garlic function of the EasySpin MATLAB package.^54^

#### Infrared Spectroscopy

Attenuated total reflection infrared spectroscopy (ATR-IR) measurements were performed in a single-reflection PIKE ATR-IR setup and a customized ATR-cell using a Si prism with an angle of incidence of 60°. A graphite suspension (0.01 g mL^–1^ in isopropanol) was spin-coated onto the Si prism. ATR-IR spectra were recorded from 4000 to 1000 cm^–1^ with a spectral resolution of 4 cm^–1^ on a Bruker Vertex 70 spectrometer equipped with a photovoltaic MCT detector. Two hundred scans were co-added for one spectrum. The spectra were acquired under a constant N_2_ flow. Background spectra were acquired before every measurement and subtracted from measured spectra.

#### Quantum Chemical Calculations

Density functional theory (DFT) calculations were performed on Gaussian09 (revision D1). Geometry optimization and vibrational analysis were performed with a def2-TZVP basis set using the M06–2X functional. A correction to a 1 M standard was applied (1.9 kcal mol^–1^). Solvent effects for the geometry optimization and single-point calculations using the PCM solvation model with the dielectric constant of H_2_O (*e* = 78.4) were used. The reduction potential (*E*^0^(*V*)) with respect to the Ag/AgCl electrode was calculated using
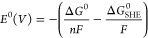
with Δ*G*^0^ being the energy difference between the reduced and oxidized molecule, *n* the number of electrons, *F* the faradaic constant (96,458.3329 s A mol^–1^), and  the absolute potential of the standard hydrogen electrode (−4.28 eV). Reaction Gibb’s free energies (Δ*G*) were calculated based on the energy difference between the two corresponding ground states. Transition state energies (Δ*G*^‡^) were calculated based on the energy difference between the transition state and the corresponding ground state.

#### Microbial Fermentation of Food Waste

Food waste (mixed bakery waste) was collected from the local grocery store. It was dehydrated in a 60 °C oven overnight (about 16 h), then crushed into powder, and kept in a −20 °C freezer before use. Microorganisms *A. awamori* (DSMZ 63272), *A. oryzae* (DSMZ 1147), and *A. succinogenes* (DSMZ 22257) were obtained from the Leibniz Institute DSMZ-German Collection of Microorganisms and Cell Cultures (Braunschweig, Germany). *A. awamori* and *A. oryzae* were utilized for the production of hydrolysis enzymes, and *A. succinogenes* was used for succinic acid fermentation, as previously reported.^39,40^

*A. awamori* and *A. oryzae* were first recovered from freeze-dried stocks by adding 0.5 mL of potato dextrose broth (Sigma-Aldrich) to dissolve each pellet and then transferring the suspension into 4.5 mL of the same medium. Next, approximately 1 mL suspension of each microorganism was plated on a potato dextrose agar (PAD) plate, *A. awamori* was incubated at 24 °C, and *A. oryzae* was incubated at 30 °C, respectively, for 7 days. The residual suspensions were stored in a −80 °C freezer as glycerol (20%) stocks.

After 7 days of growth on the PDA plates, spores of *A. awamori* and *A. oryzae* were collected by pipetting 2 mL of sterilized H_2_O to wash off the spores. The spore suspensions of *A. awamori* (dark gray color) and *A. oryzae* (light yellow color) were mixed with food waste power (10 g), respectively. The mixtures were incubated at 30 °C for 7 days to perform solid-state fermentation (SSF) for producing amylolytic and proteolytic enzymes.

Next, enzymatic hydrolysis was carried out by mixing the SSF products with food waste powder and H_2_O in a 250 mL flask, including 150 mL of H_2_O, 20 g of SSF (10 g of each microorganism), and 20 g of food waste powder. The mixture was further incubated at 55 °C, 180 rpm for 3 days. The hydrolysis product was then filtered using Whatman No.1 filter paper and centrifuged for 1 h at 10,000 rpm, followed by filtering using a 0.22 μm syringe filter, to collect the supernatant (bright yellow solution). The hydrolysis solution was kept at −20 °C before the next step.

*A. succinogenes* was first recovered from freeze-dried stocks by adding 0.5 mL of brain heart infusion broth (Sigma-Aldrich) to dissolve each pellet, and then the suspension was transferred into 4.5 mL of the same medium and incubated at 37 °C, 180 rpm overnight. Next, the bacteria culture (5 mL) was washed by centrifugation with M9 salts (Sigma-Aldrich) buffered twice to remove the culture medium, and then inoculated into 100 mL of hydrolysis solution in a serum bottle (Sigma-Aldrich), together with 10 g L^–1^ magnesium carbonate. The serum bottle was then sealed, purged with CO_2_/N_2_ mixed gas (20%: 80%, v/v) for half an hour, and fermented at 37 °C, 180 rpm for 2 days. The fermentation broth (bright orange liquid) was collected by centrifugation for 30 min at 10,000 rpm and filtered using a 0.22 μm syringe filter, kept at −20 °C before further usage.

#### Bio-Electrocatalytic Flow Tests for Conversion of Food Waste to Ethylene

Prior to the electrochemical flow tests, batch tests were conducted in a two-electrode configuration with carbon paper (graphitic) as the working electrode and Pt as the counter electrode. The reaction solution consisted of the microorganism-digested solution obtained after fermentation. A voltage of 3 V (CPE) was applied for the batch tests for a duration of 2 h.

An ElectroCell MicroFlowCell was used for the flow bio-electrochemistry experiments. The flow cell consisted of solid graphite electrodes (active area: 10 cm^2^). The flow cell was connected to a solution reservoir housing the microorganism-digested fermentation broth (containing ∼0.3 M succinic acid; pH ∼6). Prior to the tests with the real-world solution, preliminary experiments with 0.1 M aqueous succinic acid (adjusted to pH 10 with NaOH) were also carried out. The solution from the sealed reservoir was constantly circulated throughout the experiment. Similar to the batch tests, the CV scans were taken from 0 to 3 V, followed by CPE measurement at 3 V for 2 h. The solution in the reservoir was purged with N_2_ (2% CH_4_ as internal standard) prior to the experiments.

#### Product Analysis

The accumulated hydrocarbon products in the headspace were measured by an Agilent 7890A gas chromatograph equipped with a flame ionization detector (FID) and thermal conductivity detector (TCD). Splitless injection mode was applied with an inlet temperature of 120 °C, and A PLOT-MS 5A Molsieve column and an HP PLOT Q column were used for product separation, with N_2_ as the carrier gas and a constant oven temperature of 50 °C and a pressure of 16.0 psi. Gas calibration mixtures containing a known amount of the particular product were utilized to quantify the detected amount of the products. High-performance liquid chromatography (HPLC) separations were conducted with a Phenomenex Rezex 8% Ca^2+^ column at 75 °C column temperature. Samples were analyzed in the isocratic flow mode (flow rate 0.025 M H_2_SO_4_ in water, 0.5 mL min^–1^) using a Waters Breeze system equipped with refractive index (RID-2414) and diode array UV–vis (λ = 254 nm) detectors. To identify particular substances in the reaction samples, retention times were compared to those of authentic samples. Calibration was conducted with external standards. ^1^H-nuclear magnetic resonance (^1^H-NMR) spectroscopy was used to analyze gaseous products by transferring the reaction atmosphere into an evacuated Young NMR tube with *d*_6_-benzene as the solvent. NMR spectra were collected with a Bruker 400 MHz Neo Prodigy spectrometer.

## Supporting Information Available

^1^H and ^13^C NMR spectra, XRD patterns, Raman spectra, ESCA determination, XPS spectra, Koutecky–Levich plots and RRDE plots with propanoic acid, CV flow reactor, supporting tables with faradaic yields, Raman spectroscopy results, slopes of Koutecky–Levich plots, electrochemical reaction results, and conditions of quantum chemical calculations (PDF)
Electrochemical flow reactor to enhance the practicality for the continuous conversion of larger amounts of the fermentation broth (MOV)

## Supplementary Material

cs2c02689_si_001.pdf

cs2c02689_si_002.mov
