# Supplementary material for: Bio-Electrocatalytic Conversion of Food Waste to Ethylene via Succinic Acid as the Central Intermediate
Source: ACS Catal. 2022 Oct 18;12(21):13360–71. doi: 10.1021/acscatal.2c02689 (PMC9638992; doi:10.1021/acscatal.2c02689)
Supplement: Supplementary file 1 — cs2c02689_si_001.pdf [file cs2c02689_si_001.pdf]

Supporting Information

Bio-electrocatalytic conversion of food waste to ethylene via succinic acid as the central intermediate

Christian M. Pichler[a,c,d,‡] Subhajit Bhattacharjee[a,‡] Erwin Lam[a], Lin Su[a], Alberto Collauto[b], Maxie M. Roessler[b], Samuel J. Cobb[a], Vivek M. Badiani[a], Motiar Rahaman[a] and Erwin Reisner[1]*

‡ These authors contributed equally to this work

Affiliations:

[a]Yusuf Hamied Department of Chemistry, University of Cambridge, Lensfield Road, Cambridge CB2 1EW, U.K.

[b]Department of Chemistry and Centre for Puls EPR Spectroscopy (PEPR), Imperial College London, Molecular Sciences Research Hub, White City Campus, Wood Lane, London, W12 0BZ, U.K.

Current Affiliations:

[c]Institute for Applied Physics, Vienna University of Technology, A-1040 Vienna, Austria

[d]Centre of Electrochemical and Surface Technology, Viktor Kaplan Straße 2, A-2700 Wiener Neustadt, Austria

AUTHOR INFORMATION

Corresponding Author:

*Erwin Reisner, reisner@ch.cam.ac.uk

Supporting Figures

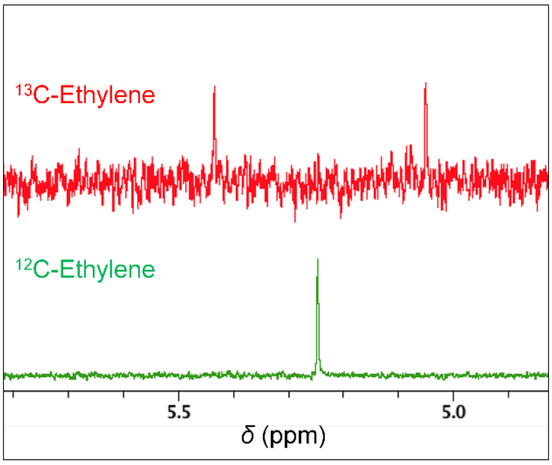

Figure S1. ^{1}H-NMR spectra after electrochemical experiments with ^{13}C-labelled succinic acid as the electrolyte with d_6-benzene as solvent . The ^{13}C-labelled succinic acid produces ^{13}C-labelled ethylene (red) after the reaction, coupling constant, 5.45 – 5.05 ppm J = 160 Hz. The green spectra is the ^{12}C-ethylene from an authentic ^{12}C-succinic acid reference, 5.25 ppm . Conditions: carbon-paper electrode, 2.8 V vs. RHE applied potential, 2 h.

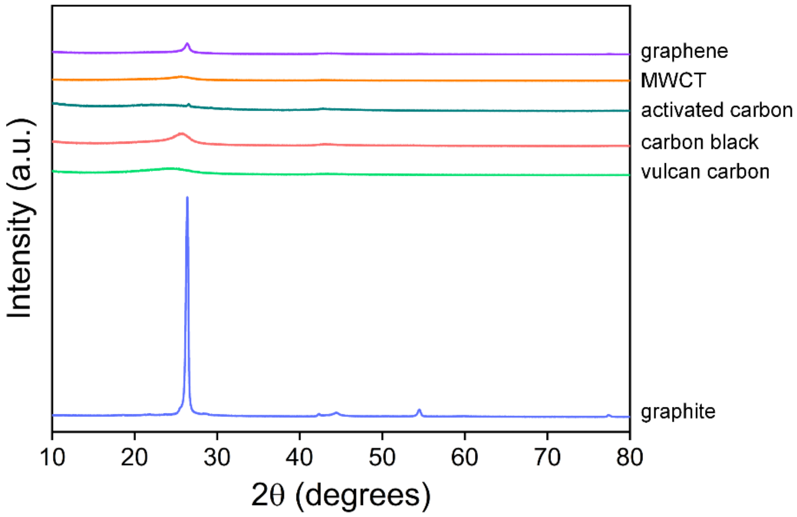

Figure S2. PXRD patterns of the different carbon materials. The distinctive graphite peak can be seen at 27°.

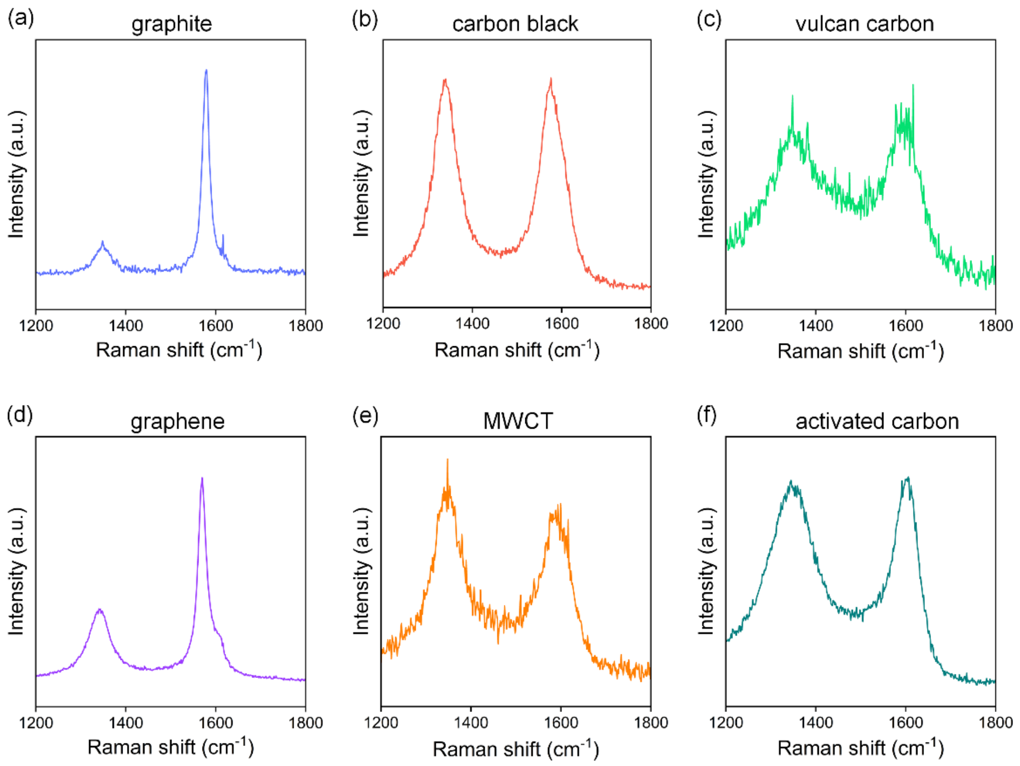

Figure S3. Raman spectra of (a) graphite, (b) carbon black, (c) vulcan carbon, (d) graphene, (e) MWCT, and (f) activated carbon with the G band at 1570 cm^{-1} (ordered, graphite-like structure) and the D band at 1370 cm^{-1} (unordered structure).

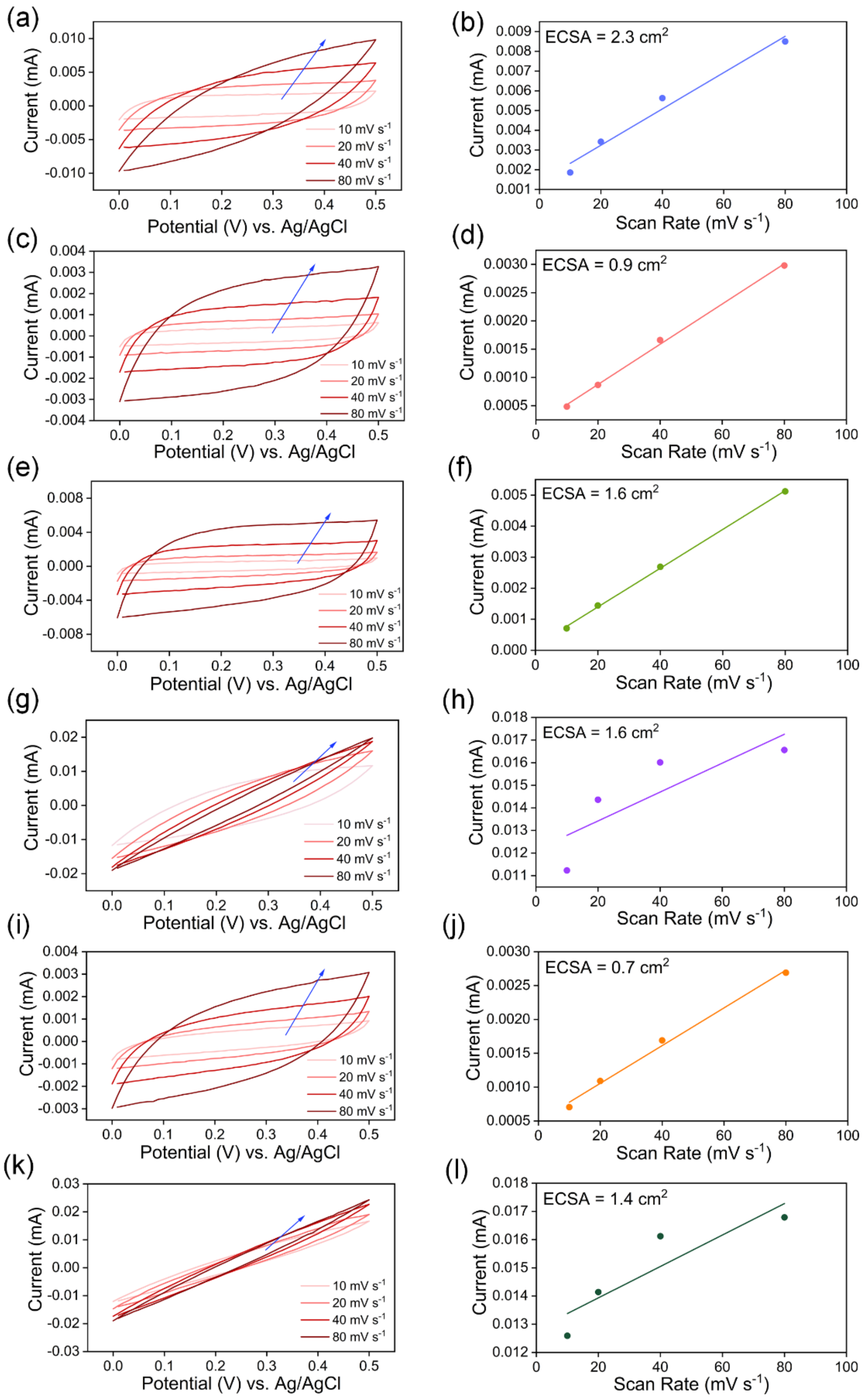

Figure S4. Determination of electrochemical surface area (ESCA) for the different carbon materials. (a,c,e,g,i,k) CV plots taken in the range of 0 – 0.5 V vs. Ag/AgCl (0.8 – 1.3 V vs. RHE) (scan rate 0.01 V s^{-1}) for (a) graphite, (c) carbon black, (e) vulcan carbon, (g) graphene, (i) MWCT and (j) activated carbon. (b,d,f,h,j,l) The corresponding current vs. scan rate plots for determination of C_{dl}.

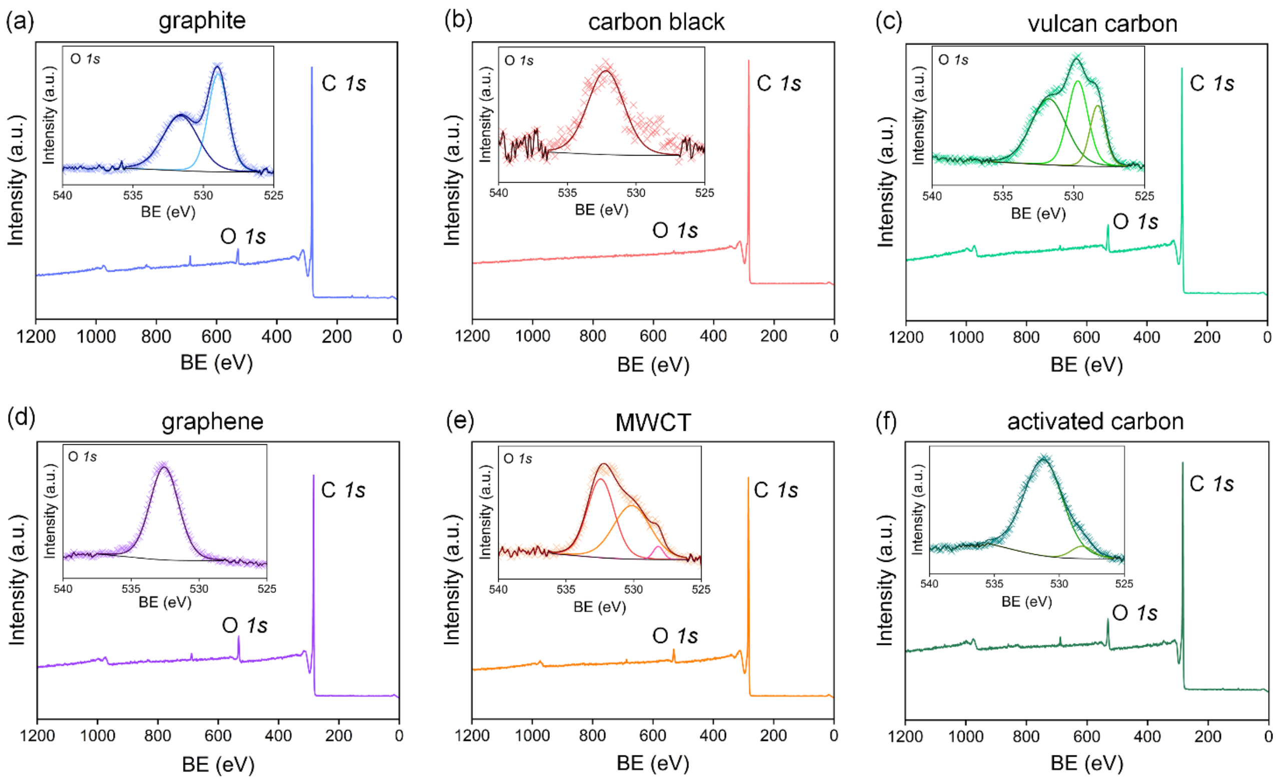

Figure S5. Survey XPS spectra of various carbon materials with inset showing O *1s* region (530 and 533 eV): (a) graphite, (b) carbon black, (c) vulcan carbon, (d) graphene, (e) MWCT and (f) activated carbon. The C-O and chemically bound OH species show peaks at ~531 and 528 eV, respectively (Belova, A. I.; Kwabi, D. G.; Yashina, L. V.; Shao-Horn, Y.; Itkis, D. M. *J. Phys. Chem. C* **2017**, *121*, 1569–1577).

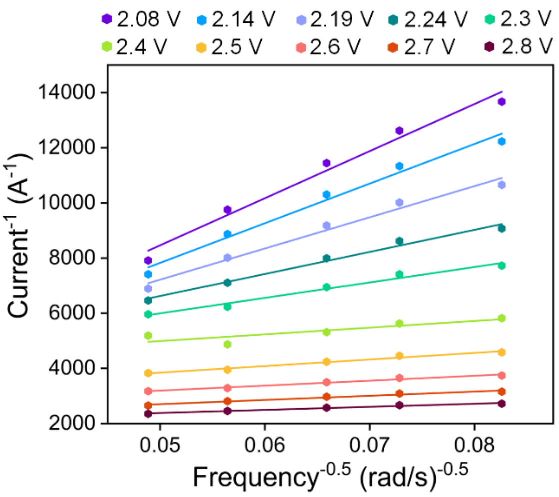

Figure S6. Koutecky-Levich plots (5 μL graphite suspension with 20 mg graphite mL^{-1} ethanol and 75 μL of 2 wt.% Nafion solution mL^{-1} ethanol drop-cast on Au-RDE) in 0.01 M propanoic acid set to pH 10 with NaOH. The potentials indicated are vs. RHE.

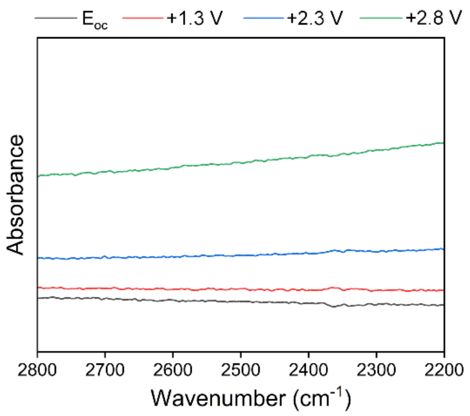

Figure S7 *in-situ* IR measurements at different applied potentials (vs. RHE) in the absence of succinic acid substrate (pH 10).

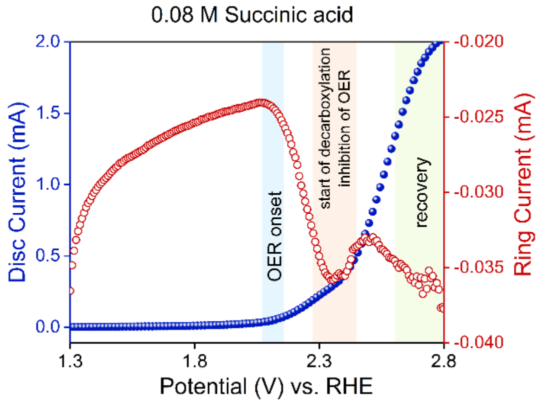

Figure S8. RRDE experiments with a glassy carbon disc (5 mm diameter, Voltage screen 1.3 – 2.8 V vs. RHE) and Pt ring (constant potential: 0.1 V vs. RHE) with 0.08 M aqueous succinic acid set to pH 10 with NaOH (rotation speed: 600 rpm).

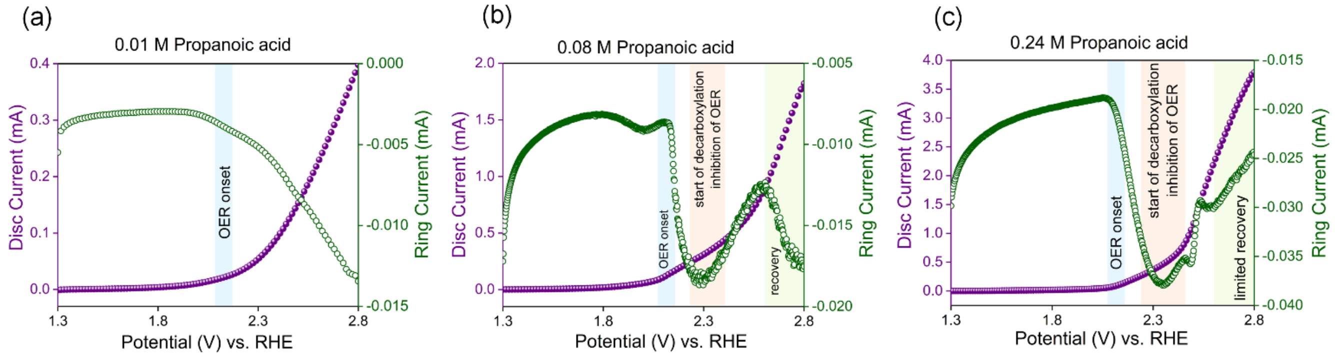

Figure S9. RRDE experiments with a glassy carbon disc (5 mm diameter, Voltage screen 1.3 – 2.8 V vs. RHE) and Pt ring (constant potential: 0.1 V vs. RHE) with (a) 0.01 M, (b) 0.08 M and (c) 0.24 M aqueous propanoic acid set to pH 10 with NaOH (rotation speed: 600 rpm).

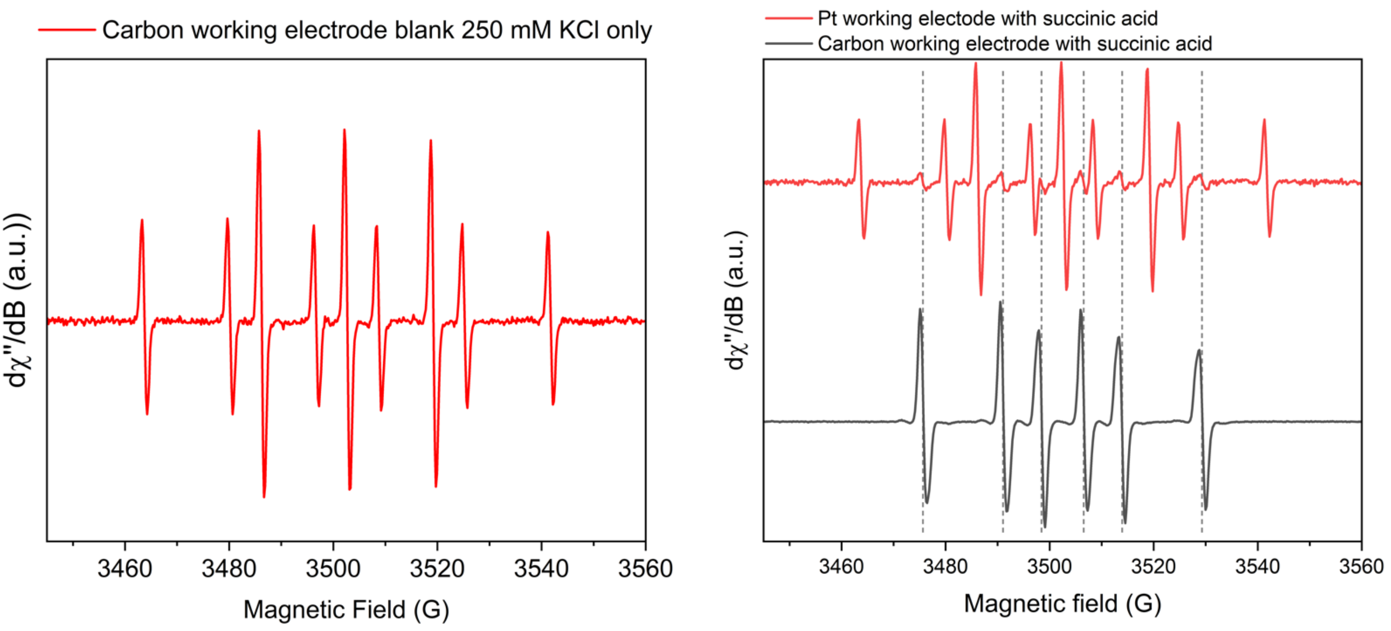

Figure S10. EPR control experiments. (left) A spin trapping experiment with the carbon working electrode and no succinic acid substrate yields a signal corresponding to DMPO-H species (a_N = 1.66 mT, a_H = 2.26 mT, 2 equivalent protons). (right) Using a Pt-wire electrode with succinic acid substrate compared with carbon electrode and succinic acid substrate (90 mM succinic acid in 150 mM NaOH in both cases) it can be seen that in the former case no succinic acid intermediate but rather the DMPO-H species is formed and that the carbon working electrode is required for the production of the trapped carbon-centred radicals.

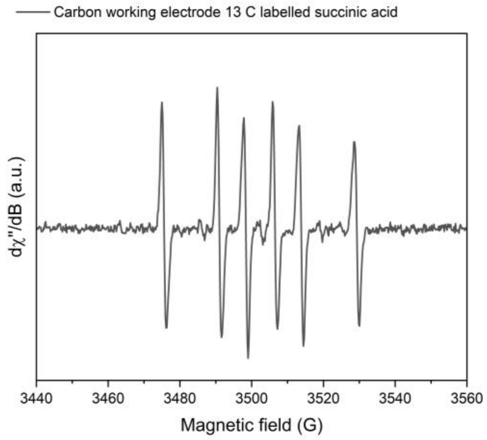

Figure S11. EPR control experiments ^{13}C labelled succinic acid (only C_2 and C_3 labelled)

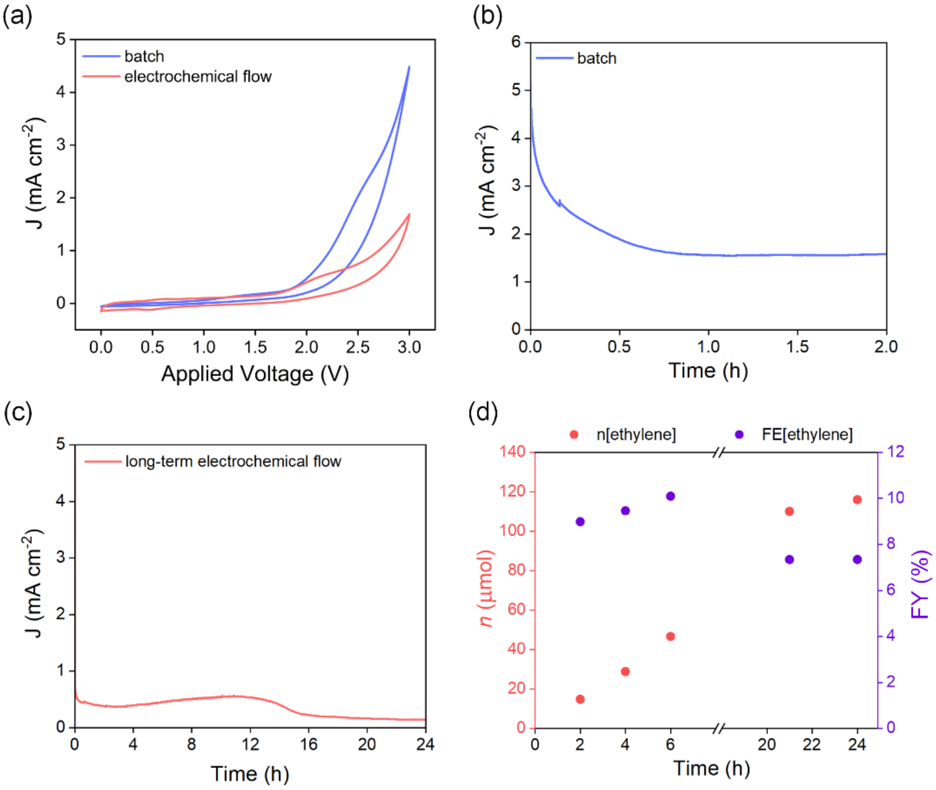

Figure S12. Representative (a) CV scans and (b, c) CPE traces for the bio-electrocatalytic process in batch (b) and electrochemical flow setup (c) using microorganism digested food waste solution. (d) Representative time-dependent ethylene evolution and corresponding FYs during electrochemical flow experiment using microorganism digested food waste solution. Conditions: CV scan rate 50 mV s^{-1}; CPE applied voltage 3 V; room temperature.

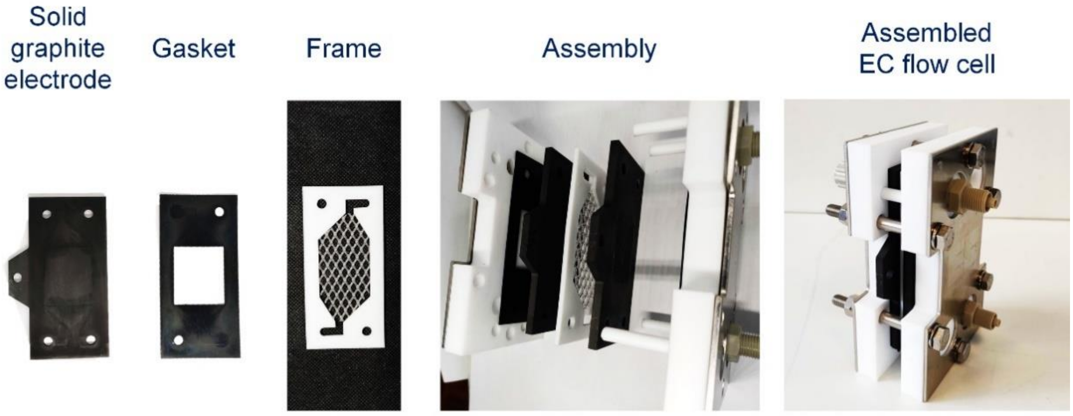

Figure S13. Individual components and assembly of the EC flow cell.

Supporting Tables

Table S1: FYs and molar yields of various carbon materials for electrochemical conversion of succinic or propanoic acid to ethylene (0.08 M acid in H_2O, set to pH 10 with NaOH, applied potential: 2.2 V vs. RHE; carbon material dispersed in ethanol, 20 mg mL^{-1} with 75 µL of 2 wt.% Nafion solution mL^{-1} ethanol and drop-casted on 1 mm diameter Au-RDE; 600 rpm).

**Material**	**FY of ethylene (%) [Substrate: succinic acid]**	**Ethylene yield (µmol) [Substrate: succinic acid]**	**FY of ethylene (%) [Substrate: propanoic acid]**	**Ethylene yield (µmol) [Substrate: propanoic acid]**
graphite	27.5 ± 8.1	1.09 ± 0.41	27.4 ± 2.4	1.83 ± 0.24
carbon black	7.4 ± 2.1	0.05 ± 0.03	18.9 ± 1.1	0.50 ± 0.002
vulcan carbon	3.6 ± 0.8	0.06 ± 0.04	10.2 ± 2.9	0.29 ± 0.03
graphene	5.3 ± 3.4	0.12 ± 0.02	4.8 ± 1.0	0.11 ± 0.04
MWCT	3.7 ± 2.4	0.04 ± 0.01	3.4 ± 0.9	0.07 ± 0.02
activated carbon	2.0 ± 0.2	0.02 ± 0.001	3.6 ± 2.0	0.09 ± 0.05

Table S2. Material characterization of different carbon materials used as electrodes. The G:D band ratio was determined by Raman spectroscopy, BET surface area and pore volume was determined by N_2-physisorption, the ESCA measurements were performed electrochemically and the oxygen content was obtained by XPS analysis.

**Material**	**G:D band ratio**	**BET surface area (m^2 g^{-1})**	**Pore volume (cm^3 g^{-1})**	**ECSA (cm^2)**	**O content (%)**
graphite	6.0	9.8	0.04	2.3	3.0
carbon black	1.3	73.9	0.26	0.9	0.3
vulcan carbon	0.9	216.9	0.48	1.6	5.0
graphene	1.2	558.8	1.02	1.6	4.0
MWCT	1.0	250.6	2.77	0.7	2.7
activated carbon	0.8	667.7	0.58	1.4	5.6

Table S3. Slopes (= $1/B_L$) for the Koutecky-Levich plots

Voltage (V)	0.01 M Succinic acid (slope)	0.01 M Propanoic acid (slope)	Blank H_2O (slope)
2.0	8053	11177	8468
1.9	9040	15192	5723
1.8	13051	17910	7784
1.7	17265	23839	7558
1.6	26343	24289	7255
1.5	45929	56120	7306
1.45	83698	80057	6174
1.4	175128	113040	5487
1.35	379132	143698	9385
1.3	217414	170750	4890

Table S4. Electrochemical reaction results (2 electrode setup, CPE 3 V for flow tests, carbon paper working electrode and Pt counter eletctrode for batch, graphitic electrodes for working and counter electrode for flow, reaction time 2 h)

Process	Time (h)	Substrate	n[ethylene] (μmol)	*n*[ethylene] (μmol cm^{-2} h^{-1})	$FY_{ethylene}$ (%)
**Batch Test**	2	microbe-digested aq. food waste solution (succinic acid ~0.3 M; pH ~6)	21.3±13.5	5.3±3.4	9.6±4.7
**Electrochemical flow setup**	1<br>2	0.1 M pure aq. succinic acid (pH ~10)	12.9±1<br>26.1±3	1.3±0.1<br>1.3±0.2	11.1±4.9<br>11.4±5.5
**Electrochemical flow setup**	2<br>24	microbe-digested aq. food waste solution (succinic acid ~0.3 M; pH ~6)	13.9±1.3<br>94.0±31.2	0.7±0.1<br>0.4±0.1	8.1±1.2<br>5.2±3.1
**Blank tests**	2	No succinic acid substrate	0.0	0.0	0.0

Supporting Discussion

Equation 1: Equation for Koutecky-Levich Plot

$$\frac{1}{i_m} = \frac{1}{i_k} + \frac{1}{B_L \omega^{0.5}}$$

i_m : measured current

i_k : kinetic current

B_L : Levich constant given as $0.6*nFAD^{2/3}v^{-1/6}C$ (n - number of transferred electrons, F - Faradaic constant in C/mol, D - Diffusion constant cm^2/s, A - Electrode area in cm^2, v - kinematic viscosity cm^2/s, C - analyte concentration in mol/cm^3)

Equation 2: Calculation of Ethylene yield per kWh

$$y_{ethylene} = \frac{mol_t}{V \times A \times t}$$

$y_{ethylene}$: Ethylene yield per kWh utilized electricity

mol_t : Mol of ethylene formed after reaction time t

V : Applied Voltage in V

A : Average current in A over reaction time t

t : reaction time in h

Estimation of energy consumption for ethylene production:

Equation 3:

$$E_{ethylene} = \frac{U \times I \times t \times 3600 \times 10^{-6}}{p_{ethylene}}$$

$$E_{ethylene-H2\,use} = \frac{U \times I \times t \times 3600 \times 10^{-6}}{p_{ethylene}} - \frac{p_{H2} \times 120}{p_{ethylene}}$$

$E_{ethylene}$: Amount of energy input required per kg of ethylene obtained (MJ kg^{-1})

$E_{ethylene\ H2use}$: Amount of energy input required per kg of ethylene obtained considering the potential utilization of energy stored in the genertated H_2 (MJ kg^{-1})

$p_{ethylene}$: Produced amount of ethylene in the flow process in 1 h in kg (approx. 100 µmol 24 h^{-1} = $2.81*10^{-6}$ kg 24 h^{-1} see Table S4)

p_{H2} : Produced amount of H_2 in the flow process (approx. 1250 µmol 24 h^{-1} = $2.5*10^{-6}$ kg 24 h^{-1})

Energy density of H_2 in MJ kg^{-1} : 120

U : Applied Voltage in flow test (3 V)

I : Consumed current in flow tests (0.15 mA cm^{-2} → 0.0015 A for 10 cm^2 electrode area)

t : time (24 h)

Factor to convert Wh in J : 3600

Factor to convert J in MJ : 10^{-6}

Estimation of CO_2 emission for ethylene production:

Equation 4:

$$Emis_{ethylene} = \frac{1}{m_{ethylene}} \times 2 \times m_{CO2} - \frac{1}{m_{ethylene}} \times m_{succ} \times B \times CO2_{compost}$$

$Emis_{ethylene}$: CO_2 emissions in kg per kg produced ethylene

$m_{ethylene}$: molar mass of ethylene (28.05 g mol^{-1})

m_{CO2} : molar mass of ethylene (44.01 g mol^{-1})

m_{succ} : molar mass of succinic acid (118.09 g mol^{-1})

$CO2_{compost}$: Amount of CO_2 generated when composting food waste (0.37 kg CO_2 kg^{-1} food waste)[1]

B : Amount of food waste required to generate 1 kg of succinic acid in the fermentation process (3.3 kg food waste per kg succinic acid)[2]

Quantum mechanical calculations

CO_2 Charge:0 Multiplicity: 1

C	0.0006527	-0.0000650	-0.0002377
O	-0.4974183	-0.4569610	-0.9362687
O	0.4967657	0.4570260	0.9365063

Ethylene Charge:0 Multiplicity: 1

C	-0.6479387	-0.1349275	-0.0000010
C	0.6479393	0.1349275	0.0000000
H	-1.0328427	-1.0699755	0.3885960
H	-1.3740927	0.5687535	-0.3885960
H	1.3740923	-0.5687535	0.3885960
H	1.0328423	1.0699755	-0.3885950

Ethylene Charge:0 Multiplicity: 3 (biradical)

C	-0.6910508	-0.2155148	-0.0019032
C	0.6919602	0.2115562	0.0011928
H	-0.9761158	-1.2163958	-0.3072072
H	-1.4923118	0.4502732	0.2994158
H	1.3213442	0.0955732	0.8768848
H	1.1461742	0.6745082	-0.8683832

Ethylene Charge:-1 Multiplicity: 2

C	-0.7138782	-0.0038162	0.0808610
C	0.7139318	0.0044198	-0.0818180
H	-1.2649352	-0.9059592	-0.1836640
H	-1.2681772	0.9138968	-0.1137260
H	1.2673378	-0.9138882	0.1131710
H	1.2657208	0.9053468	0.1851760

Succinic acid Charge:-2 Multiplicity: 1

C	-1.9685232	-0.0009139	0.0442343
C	-0.5406592	0.5635711	0.0367313
C	0.5254748	-0.5082029	-0.1257237
H	-0.3770852	1.0748061	0.9884773
H	-0.4533762	1.3137391	-0.7505057
C	1.9774448	-0.0219939	0.0099773
H	0.4248998	-1.0125999	-1.0896487
H	0.3746178	-1.2765869	0.6378373
O	-2.1952602	-0.9595019	0.8219393
O	2.1862158	1.0304981	0.6566203
O	-2.8099182	0.5438641	-0.7111677
O	2.8561688	-0.7466789	-0.5187717

Succinic acid Charge:-1 Multiplicity: 2

C	-1.9163325	-0.0549879	-0.0814748
C	-0.5231145	0.5382191	-0.1370427

C	0.5245325	-0.5102719	0.2026132
C	1.9715455	-0.0093359	-0.0004728
H	0.3854745	-1.3910549	-0.4265578
H	0.4203335	-0.8424539	1.2364073
O	-2.7455585	0.7846371	0.4903602
O	2.1315615	1.0955821	-0.5591558
O	-2.2671035	-1.1308369	-0.5007367
O	2.8614035	-0.7840839	0.4131662
H	-0.3910165	0.9007521	-1.1584617
H	-0.4517255	1.4038351	0.5213552

Succinic acid Charge:0 Multiplicity: 3

C	-1.4318202	-1.2568710	-0.0054305
C	-0.7543893	0.0868500	0.0103985
C	0.7543868	-0.0868520	-0.0104625
H	-1.0828502	0.6265810	0.8999565
H	-1.1034943	0.6576120	-0.8514985
C	1.4318187	1.2568710	0.0053685
H	1.0828498	-0.6265770	-0.9000225
H	1.1034898	-0.6576200	0.8514325
O	-0.9090873	-2.3422740	-0.0306755
O	0.9090938	2.3422740	0.0307885
O	-2.7497803	-1.1802610	0.0116515
O	2.7497828	1.1802670	-0.0115065

Propionic acid Charge –1 Multiplicity 2

C	1.3023263	0.2319506	-0.0794928
C	0.0511913	-0.5167004	0.1703582
H	1.6123673	1.0231016	0.5875302
H	1.8144723	0.1487696	-1.0281268
C	-1.2702417	0.2721496	-0.0981238
H	-0.0168997	-0.8146294	1.2233752
H	0.0086743	-1.4322304	-0.4195398

O	-1.2223487	1.5185126	-0.0335588
O	-2.2795417	-0.4309234	-0.3224218

Propionic acid Charge 0 Multiplicity 3

C	1.2711643	0.3260472	0.0944312
C	0.0665943	-0.4997748	-0.1625528
H	1.1787833	1.3470542	0.4274632
H	2.2466843	-0.1081838	-0.0555788
C	-1.2101337	0.2611602	0.0950882
H	0.0428923	-1.4023518	0.4600752
H	0.0284293	-0.8659468	-1.1956668
O	-1.3300777	1.4013602	0.4651142
O	-2.2943367	-0.4593648	-0.1283738

Propionic acid Charge -2 Multiplicity 1

C	1.3988212	0.0287714	0.3032409
C	0.1213352	-0.4969376	-0.3921851
H	1.2224732	-0.0034776	1.3923479
H	1.4884812	1.1033484	0.0672449
C	-1.1579738	0.2521754	-0.0193181
H	-0.0277328	-1.5548686	-0.1455571
H	0.2385322	-0.4369176	-1.4807081
O	-1.4730938	1.2636244	-0.7068941
O	-1.8108428	-0.1557186	0.9818289

Transition State Succinic acid (biradical) to propionic acid

C	-2.0204827	0.1222431	0.0443051
C	-0.4946957	-0.6155179	0.0767981
C	0.5011102	0.5067431	-0.0908169
H	-0.4239688	-1.3827689	-0.6831919
H	-0.5273488	-1.0465909	1.0760481
C	1.9034872	-0.0443619	-0.0233739
H	0.3851963	1.2599321	0.6887961

H	0.3862543	1.0086071	-1.0543249
O	-2.1674258	1.0649201	0.7660361
O	2.2400723	-1.1875299	0.1416051
O	-2.6199047	-0.5672899	-0.7758749
O	2.8377063	0.8816141	-0.1660059

Transition propionic acid to ethylene

C	-1.1753232	0.3301400	-0.3593604
C	-0.1257082	-0.4235620	0.3597566
H	-1.0118122	0.7103180	-1.3558994
H	-2.1831142	0.2854610	0.0287176
C	1.3024778	0.2328790	-0.0060524
H	-0.1371362	-1.4943190	0.1481596
H	-0.1495782	-0.2927550	1.4449196
O	1.4864258	1.4146660	0.0797746
O	1.9937688	-0.7628280	-0.3400154

Supporting Movie

Movie S1: Electrochemical evolution of ethylene from microbially digested solution with flow reactor setup. Conditions: E_{cell}: 3 V, pH 6, room temperature.

Supporting References

(1) Komilis, D. P.; Ham, R. K. Carbon Dioxide and Ammonia Emissions during Composting of Mixed Paper , Yard Waste and Food Waste. *Waste Manag.* **2006**, *26*, 62–70. https://doi.org/10.1016/j.wasman.2004.12.020.

(2) Zhang, A. Y. Z.; Sun, Z.; Leung, C. C. J.; Han, W.; Lau, K. Y.; Li, M.; Lin, C. S. K. Valorisation of Bakery Waste for Succinic Acid Production. *Green Chem.* **2013**, *15* (3), 690–695. https://doi.org/10.1039/c2gc36518a.

End of Supporting Information
